# Molecular insights into the interaction between a disordered protein and a folded RNA

**DOI:** 10.1073/pnas.2409139121

**Published:** 2024-11-26

**Authors:** Rishav Mitra, Emery T. Usher, Selin Dedeoğlu, Matthew J. Crotteau, Olivia A. Fraser, Neela H. Yennawar, Varun V. Gadkari, Brandon T. Ruotolo, Alex S. Holehouse, Loïc Salmon, Scott A. Showalter, James C. A. Bardwell

**Affiliations:** ^a^HHMI, University of Michigan, Ann Arbor, MI 48109; ^b^Department of Molecular, Cellular, and Developmental Biology, University of Michigan, Ann Arbor, MI 48109; ^c^Department of Biochemistry and Molecular Biophysics, Washington University School of Medicine, St. Louis, MO 63110; ^d^Center for Biomolecular Condensates, Washington University in St. Louis, St. Louis, MO 63110; ^e^Centre de Résonance Magnétique Nucléaire à Très Hauts Champs, UMR 5082, CNRS, Ecole Normale Supérieure de Lyon, Université Claude Bernard Lyon 1, Université de Lyon, Villeurbanne 69100, France; ^f^Department of Biochemistry and Molecular Biology, The Pennsylvania State University, University Park, PA 16802; ^g^The Huck Institutes of the Life Sciences, The Pennsylvania State University, University Park, PA 16802; ^h^Department of Chemistry, University of Michigan, Ann Arbor, MI 48109; ^i^Department of Chemistry, The Pennsylvania State University, University Park, PA 16802

**Keywords:** disordered proteins, RNA binding proteins, molecular condensates

## Abstract

Intrinsically disordered protein regions (IDRs) are well established as contributors to intermolecular interactions and the formation of biomolecular condensates. In particular, RNA-binding proteins (RBPs) often harbor IDRs in addition to folded RNA-binding domains that contribute to RBP function. To understand the dynamic interactions of an IDR–RNA complex, we characterized the RNA-binding features of a small (68 residues), positively charged IDR-containing protein, Small ERDK-Rich Factor (SERF). At high concentrations, SERF and RNA undergo charge-driven associative phase separation to form a protein- and RNA-rich dense phase. A key advantage of this model system is that this threshold for demixing is sufficiently high that we could use solution-state biophysical methods to interrogate the stoichiometric complexes of SERF with RNA in the one-phase regime. Herein, we describe our comprehensive characterization of SERF alone and in complex with a small fragment of the HIV-1 Trans-Activation Response (TAR) RNA with complementary biophysical methods and molecular simulations. We find that this binding event is not accompanied by the acquisition of structure by either molecule; however, we see evidence for a modest global compaction of the SERF ensemble when bound to RNA. This behavior likely reflects attenuated charge repulsion within SERF via binding to the polyanionic RNA and provides a rationale for the higher-order assembly of SERF in the context of RNA. We envision that the SERF–RNA system will lower the barrier to accessing the details that support IDR–RNA interactions and likewise deepen our understanding of the role of IDR–RNA contacts in complex formation and liquid–liquid phase separation.

## Significance

Subcellular organization through the formation of biomolecular condensates has emerged as an important contributor to myriad cellular functions, with implications in homeostasis, stress response, and disease. To understand the general and specific principles that support condensate formation, we must interrogate the interactions and assembly of their constituent biomolecules. To this end, this study introduces a simple model system composed of a small, disordered protein and small RNA that undergo charge-driven, associative phase separation. In addition to extensive biophysical characterization of these molecules and their complex, we also generate insights into mode of interaction and assembly between an unstructured protein and a structured RNA.

Intermolecular interactions underscore many critical functions as a means to interpret, transmit, and respond to numerous and diverse signals in a cell. Among the proteins involved in this so-called “information processing,” many harbor intrinsically disordered regions (IDRs) that mediate interactions even though they lack stable tertiary structures (1). Although they have roles in virtually every cellular process, IDRs have emerged as important determinants for proteins binding to RNA (2–4). Previously, RNA-binding proteins (RBPs) were assigned as such based on the presence of a structured RNA-binding domain (5), but recent large-scale interactome studies illuminated many “unconventional” RBPs with IDRs that can directly engage RNA (5–7). Such RBPs may leverage both folded and disordered RNA-interaction modules in concert to bind RNA with tailored affinity or specificity (3). Moreover, IDR-mediated interactions are not limited to binary complexes; many IDR-containing RBPs undergo higher-order assembly with RNA to form biomolecular condensates (8).

Biomolecular condensates are nonstoichiometric assemblies of biomolecules that concentrate specific components while excluding others (8). These multicomponent cellular bodies play key roles in the spatiotemporal regulation of biological processes in space and time (8, 9). While condensates can form through various mechanisms, the physics of phase separation offers a parsimonious explanation for their formation, behavior, and dissolution (10–12). Condensates often contain protein and RNA, and both can play key roles in determining condensate formation and function (13–16). The importance of IDRs and RNA in the context of condensate stems from their inherent tendency to form multivalent interactions (17). This is especially true for polycationic disordered regions, which interact directly with polyanionic RNA (18–21). As such, charge-mediated RNA–protein interactions are central in determining condensate assembly, regulation, and disassembly. So far, high-resolution biophysical investigation into the intermolecular interactions between IDRs and RNA molecules that underlie condensate formation have largely been limited to artificial peptides and simple homopolymeric RNA species which have provided simple, though not particularly physiological systems to elucidate basic physical principles (19–24).

Several practical and technical barriers have historically impeded high-resolution, quantitative characterization of IDR–RNA complexes. First, the structural heterogeneity of IDRs (and some RNAs) generally precludes classical structural interrogation methods (i.e., X-ray crystallography); therefore, we must rely on solution-state ensemble methods. Second, many of the “classical” RBPs, such as FUS, hnRNPA1, and G3BP1, are large, often aggregation-prone, and have multidomain architecture, such that the residue-level details of RNA binding are challenging to obtain (25–27). Similarly, endogenous RNA molecules can be kilobases in length, which can pose a challenge to accessing high-resolution structural and dynamic information of the molecule (8). Finally, at the intersection of the two aforementioned barriers, many of the techniques used to characterize IDRs and their complexes, such as NMR spectroscopy or small angle X-ray scattering (SAXS), require relatively high concentrations of biomolecules. For large, multidomain RBPs, such a concentration regime may be inaccessible for RBP-RNA partners that readily demix and, hence, preclude the study of stoichiometric complexes.

**Fig. 1.**
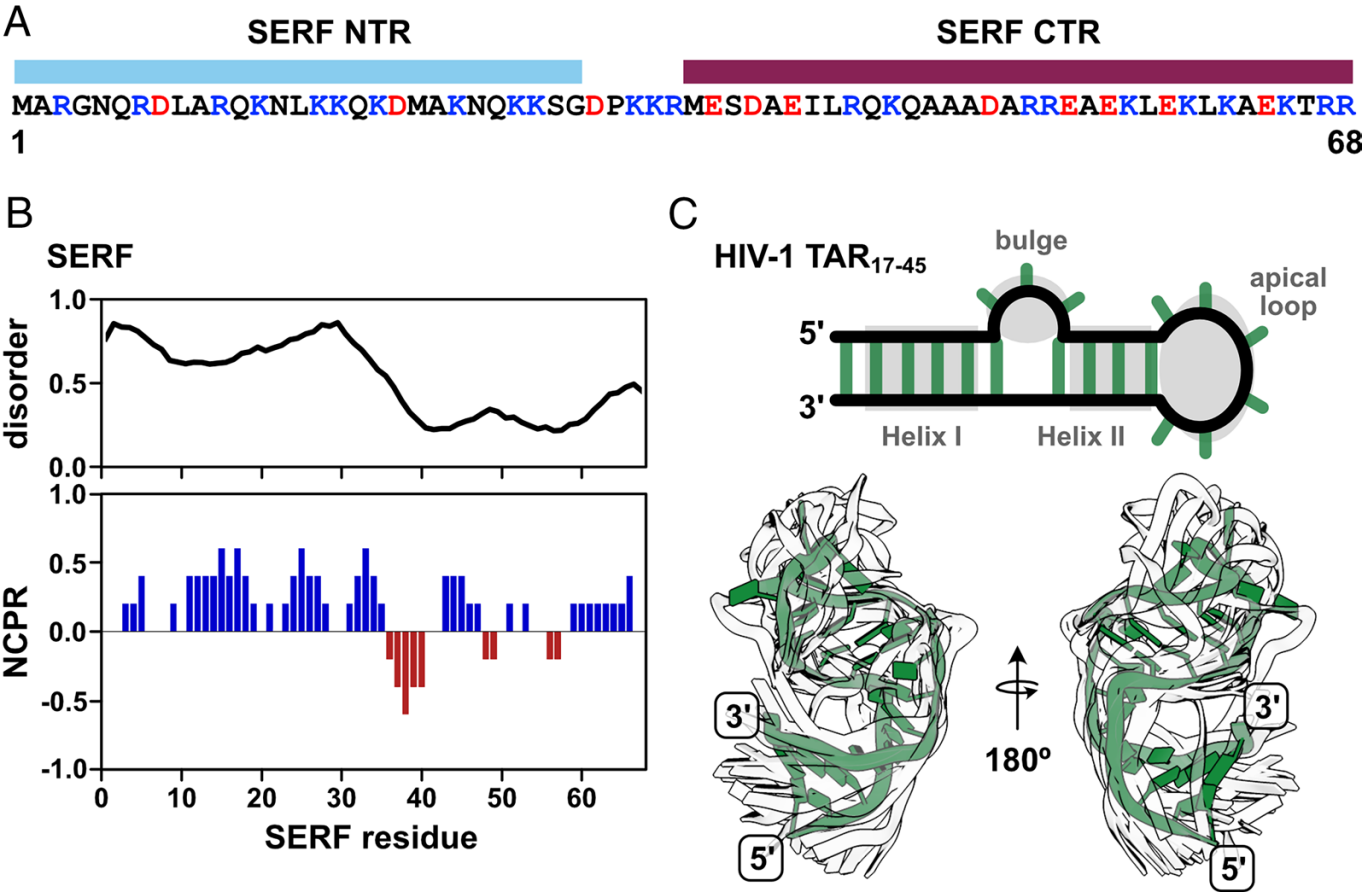
SERF is a highly positively charged disordered protein that can bind RNA. (*A*) Sequence of *S. cerevisiae* SERF with domain annotations (NTR; CTD). Positive and negative amino acids are colored in blue and red, respectively. (*B*) Per-residue disorder scores from Metapredict (35) (*Top*) and SERF net charge per residue (NCPR) calculated with CIDER using a window size of five (36). (*C*) Diagram of the fragment of the HIV-1 TAR RNA used in this study (nucleotides 17-45) (*Top*). TAR features of interest are shown in gray. Helix I and Helix II are also referenced as “upper” and “lower” helices, respectively. The *Bottom* panel shows the alignment of TAR conformers from the NMR solution structure (PDB ID: 1ANR) (37). One conformer is shown in green for clarity; the other 19 are shown as white outlines to illustrate TAR dynamics.

In this work, we sought to circumvent these challenges by investigating a pair of biomolecules in which IDR and RNA properties in the bound and unbound states could be directly interrogated by experiments and simulations. The ideal IDR–RNA system should undergo phase separation at sufficiently high concentrations such that the stoichiometric complex can be studied in the absence of condensate formation. In addition, both molecules should be relatively small (i.e., single domain) in order to make spectroscopic interpretation more straightforward. To this end, we selected the 68-residue, intrinsically disordered *Saccharomyces cerevisiae* protein, Small ERDK-Rich Factor (SERF) (UniProt ID: YDL085C-A) (Fig. 1*A*). SERF is highly charged with a bias toward positive residues and is predicted to be primarily intrinsically disordered (Fig. 1*B*). Structural studies of the *Caenorhabditis elegans* SERF homolog, MOAG-4, showed that the C-terminal region (CTR) of the protein adopts an α-helix, whereas the N-terminal region (NTR) is more structurally heterogeneous (28). Recent work showed that the human SERF protein partitions to nucleoli and can facilitate the nucleolar incorporation of fluorescently labeled RNA (29). For the minimal RNA component, we selected the apical region of the Trans-Activation Response element (TAR) RNA from HIV type-1 (HIV-1). TAR is among the best-studied helix–junction–helix motifs and, importantly, moves us beyond the study of homopolymeric RNAs (30, 31). This 29-nucleotide RNA fragment (depicted in Fig. 1*C*) contains two A-form helical stems (Helix I and Helix II), a trinucleotide bulge, and a hexanucleotide apical loop, which collectively undergo complex dynamic motions over a broad range of timescales (31). Extensive prior research into TAR and its interactions in the context of virulence equips us with a wealth of information about its structural and dynamic properties (32–34).

Our work establishes that SERF and TAR primarily form a binary complex at concentrations suitable for biophysical characterization (<0.5 mM) and can form biomolecular condensates in vitro above these concentrations or upon addition of crowding agents. In the SERF:TAR complex, neither SERF nor TAR undergo major structural rearrangements; SERF retains its conformational flexibility, while TAR similarly preserves its base-paired secondary structure. Through extensive biophysical characterization of the protein, RNA, and their complex, this study sheds light on the driving forces behind the interactions of a disordered protein with a folded RNA that may be extended to more complicated, multidomain RBPs.

## Results

### Conformational Properties of SERF.

Prior to investigating the molecular mechanisms of SERF–RNA interaction, we first characterized the SERF protein in isolation using multiple biophysical methods. We used solution NMR spectroscopy to obtain residue-level information on the structural and dynamic features of the SERF conformational ensemble. Like many disordered proteins, SERF shows limited signal dispersion in its ^1^H,^15^N-HSQC spectrum (*SI Appendix*, Fig. S1*A*), which poses a challenge for unambiguous resonance assignments and quantitative structural characterization (38, 39). Therefore, we turned to ^13^C direct-detect NMR spectroscopy, which offers improved resolution and reduced peak overlap for IDRs compared to proton-based detection (40, 41). The ^13^C, ^15^N-CON spectrum of SERF shows excellent chemical shift dispersion and enabled us to obtain backbone and sidechain assignments for each SERF residue (*SI Appendix*, Fig. S1*B* and Table S1).

**Fig. 2.**
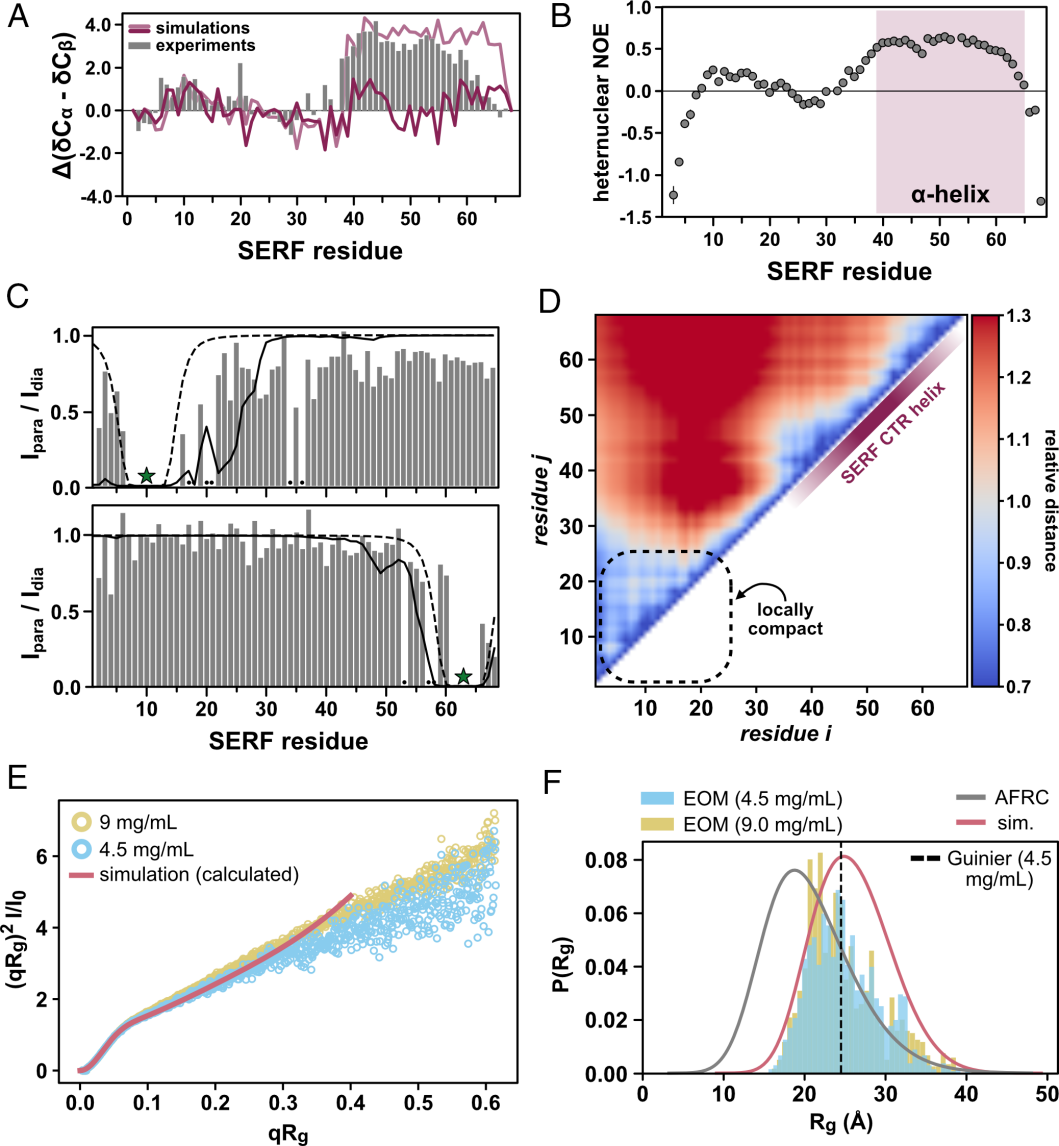
SERF is globally disordered amid regions of transient structure. (*A*) Secondary structure of SERF calculated from NMR chemical shifts (gray bars). Positive values indicate regions of alpha helix; negative values correspond to beta strand/extended regions. Average alpha helix character of SERF determined using backcalculated chemical shifts from all-atom simulations is shown as a dark pink trace. For comparison, backcalculated chemical shifts from simulations in which CTR of SERF was fixed as a helix are shown in light pink. (*B*) {^1^H}−^15^N hetNOE profile of SERF. The error bars reflect the SD from the mean of three hetNOE measurements. Error bars that were smaller than the size of the data marker are not shown. The pink shaded region reflects the location of the CTR α-helix, for which we observe the expected hetNOE values (around ~0.6). (*C*) PRE NMR profiles for SERF containing PRE labels at residues 10 (*Top*) or 63 (*Bottom*). Gray bars reflect PRE measurements: missing/unassignable resonances are denoted with a black dot. The solid line is the PRE profile calculated from all-atom simulations and the dashed line is the theoretical profile for a Flory Random Coil (null model). The star indicates the site of MTSSL attachment in PRE NMR experiments. (*D*) Normalized distance map of the SERF ensemble from simulations. Interresidue (C_α_–C_α_) distances were measured per frame and averaged; the *relative distance* is presented. Intramolecular distances relative to the AFRC model are presented such that red is more expanded than AFRC and blue is more compact. (*E*) Dimensionless Kratky transformation of scattering data. FoXS was used to calculate the average scattering profile from all-atom simulations (43). (*F*) Radius of gyration (R_g_) probability distributions for SERF from SAXS experiments and simulations. The dashed vertical line marks the R_g_ determined from the Guinier approximation. EOM distributions for two different SERF concentrations are shown as bars colored in blue or yellow. The gray distribution is that of the AFRC model for the SERF sequence and the pink trace is the distribution of R_g_ values from all-atom SERF simulations. For comparison, the R_g_ distribution from 14,250 frames was normalized by kernel density estimate.

From the disorder prediction profile (Fig. 1*B*) and an earlier structural study on *S. cerevisiae.* SERF, we expected to observe some structural features in the CTR in an otherwise disordered protein (39). To this end, we calculated per-residue secondary structure propensities from the deviations of the measured C_α_ and C_β_ chemical shifts referenced against theoretical SERF chemical shifts assuming a random coil (42). Consistent with earlier findings from ^1^H-detected NMR experiments on SERF, we observe α-helical character between residues 38 and 60 (Fig. 2*A*) (39). This conclusion is further supported by heteronuclear NOE (hetNOE) measurements, which yielded systematically higher values (~0.6) for the CTR helix compared to the rest of protein (Fig. 2*B*). The NTR of SERF lacks secondary structure aside from a short α-helical stretch near residues 5 to 15; the differences in magnitude between the N- and C-terminal helical regions suggest that the helix in the NTR is less populated relative to the helix in the CTR (Fig. 2*A*).

To further explore the local and global conformational features of the SERF ensemble, we performed all-atom Monte Carlo simulations of SERF using the CAMPARI/ABSINTH simulation package with OPLS-AA/L force field parameters (44). This simulation strategy has been applied to several disordered protein systems with good agreement with in vitro measurements (45–48). We observe qualitative agreement between the experimental secondary structure profile and that backcalculated from SERF simulations (*Materials and Methods*) (Fig. 2*A* and *SI Appendix*, Fig. S1 *C* and *D*). The simulations capture the modest helical region in NTR but fail to reproduce the more populated helix in the CTR. SERF simulations show high helical character near residues 40 to 45 and toward the distal C terminus (residues 55 to 65), but the intervening stretch of residues is much less helical compared to experiments (Fig. 2*A* and *SI Appendix*, Fig. S1*D*). Possible explanations for this discrepancy are detailed in the simulation *Materials and Methods*.

To evaluate the local conformational properties of SERF, we next measured ^15^N spin relaxation using ^13^C direct-detect NMR methods optimized for IDRs (49). The T_1_ and T_2_ relaxation times are sensitive to both global chain reorientation and local backbone motions that occur on the picosecond-nanosecond timescale (50). The T_1_ profile for SERF is largely featureless with relaxation times typical for a small IDR (*SI Appendix*, Fig. S1*E*) (49). Fast molecular rearrangements, like those that are characteristic of unstructured protein regions, generally confer longer T_2_ times as compared to more rigid, structured regions. Compared to the NTR, the T_2_ values in the CTR of SERF are depressed (*SI Appendix*, Fig. S1*F*), likely reflecting the high contact density between spin systems arising from the α-helical structure. The marked differences in T_2_ times between the two halves of SERF suggest that the molecular motions of these regions are decorrelated (*SI Appendix*, Fig. S1*F*).

To complement residue-level insights into the SERF ensemble, we examined the global conformational properties of SERF. Despite the lack of a stable tertiary structure, transient tertiary contacts and sparsely sampled compact conformations often contribute to IDR ensembles (51, 52). Thus, we asked whether transient tertiary contacts between the NTRs and CTRs of SERF contribute to its ensemble behavior. Paramagnetic relaxation enhancement (PRE) NMR measurements are highly sensitive to sparsely populated conformations with long-range contacts and can provide site-resolved information about such interactions (53). We labeled one of two exogenous cysteines on SERF at either residue 10 or 63 with the nitroxide spin label, (1-Oxyl-2,2,5,5-tetramethylpyrroline-3-methyl) methanethiosulfonate (MTSSL), and then measured PREs using ^13^C direct-detect NMR experiments. In this method, resonances for residues within ~20 Å of the labeled residue are expected to decrease in intensity due to enhanced relaxation induced by the unpaired electron on MTSSL (54).

Our PRE results represented as the ratio of peak intensities in the paramagnetic and diamagnetic measurements in Fig. 2*C*, indicate minimal long-range interactions in the ensemble; instead, PRE occurs almost exclusively around the labeling site (Fig. 2*C*). This conclusion is supported by PRE profiles calculated from all-atom simulations of SERF. Based on the width of the PRE basin flanking the N-terminal spin label, the first ~20 residues of SERF appear to be more compact than expected for a random coil model (Fig. 2 *C*, *Top*). Given the number and spacing of basic residues—and the paucity of acidic residues—it is unlikely that the NTR compaction is driven by electrostatic interactions. Rather, we propose that an interplay of transient helical character (Fig. 2*A*) and polar contacts mediated by the five Asn and Gln side chains drive the observed local compactness.

Distance maps calculated from simulations quantify ensemble-averaged interresidue distances, normalized by the distance expected for a random coil (Fig. 2*D*) (*Materials and Methods*). The distance map reflects the modest relative compactness in the NTR that we also observed in the experimental PRE profile for this region (Fig. 2*C*) and similarly suggests that SERF lacks long-range intramolecular contacts. Collectively, the spin relaxation measurements, PRE experiments, and all-atom simulations show that SERF is primarily unstructured, but contains elements of local structure and/or compactness that bias its global ensemble away from that of a random coil.

We next used small-angle X-ray scattering (SAXS) to quantify the global ensemble properties of SERF. SAXS reports on the size [reported as radius of gyration (R_g_)] and shape of a molecule based on the features of the scattering pattern (55). The scattering profiles obtained from two different concentrations of SERF are consistent with a primarily disordered protein. Further, the scattering profile calculated from all-atom simulations shows excellent agreement with both experimental datasets (Fig. 2*E* and *SI Appendix*, Fig. S2 *A*–*C*). We determined the ensemble-averaged R_g_ using the Guinier approximation, ensemble optimization method (EOM), and the Molecular Form Factor (MFF) of Riback et al. (*Materials and Methods*) (56–58). These different approaches showed good agreement with each other, with average Rg values of 24.5 ± 0.2 Å (Guinier), 25.2 Å (EOM), and 23.9 ± 0.1 Å (MFF). These are also in good agreement with Rg values calculated from all-atom simulations (Rg = 25.9 Å) and from the deep learning-based predictor, ALBATROSS (Rg = 24.8 Å) (*SI Appendix*, Table S2) (59). Compared to a null model that assumes random coil polymer behavior, which predicts an average Rg of 20.8 Å, the SERF ensemble appears to be somewhat expanded based on both simulations and experiments (Fig. 2*F*) (60). Deviation from the random coil model likely arises from the high fraction of charged residues in SERF, through which electrostatic repulsion could drive ensemble expansion in a solution with modest ionic strength (such as the buffers used in our experiments). Still, the distribution of sizes that SERF can access is additionally biased by areas of local compactness (i.e., residues 5 to 25) and secondary structure elements (i.e., helix in the CTR).

### Dynamic SERF–TAR Complexes Are Globally Compact.

**Fig. 3.**
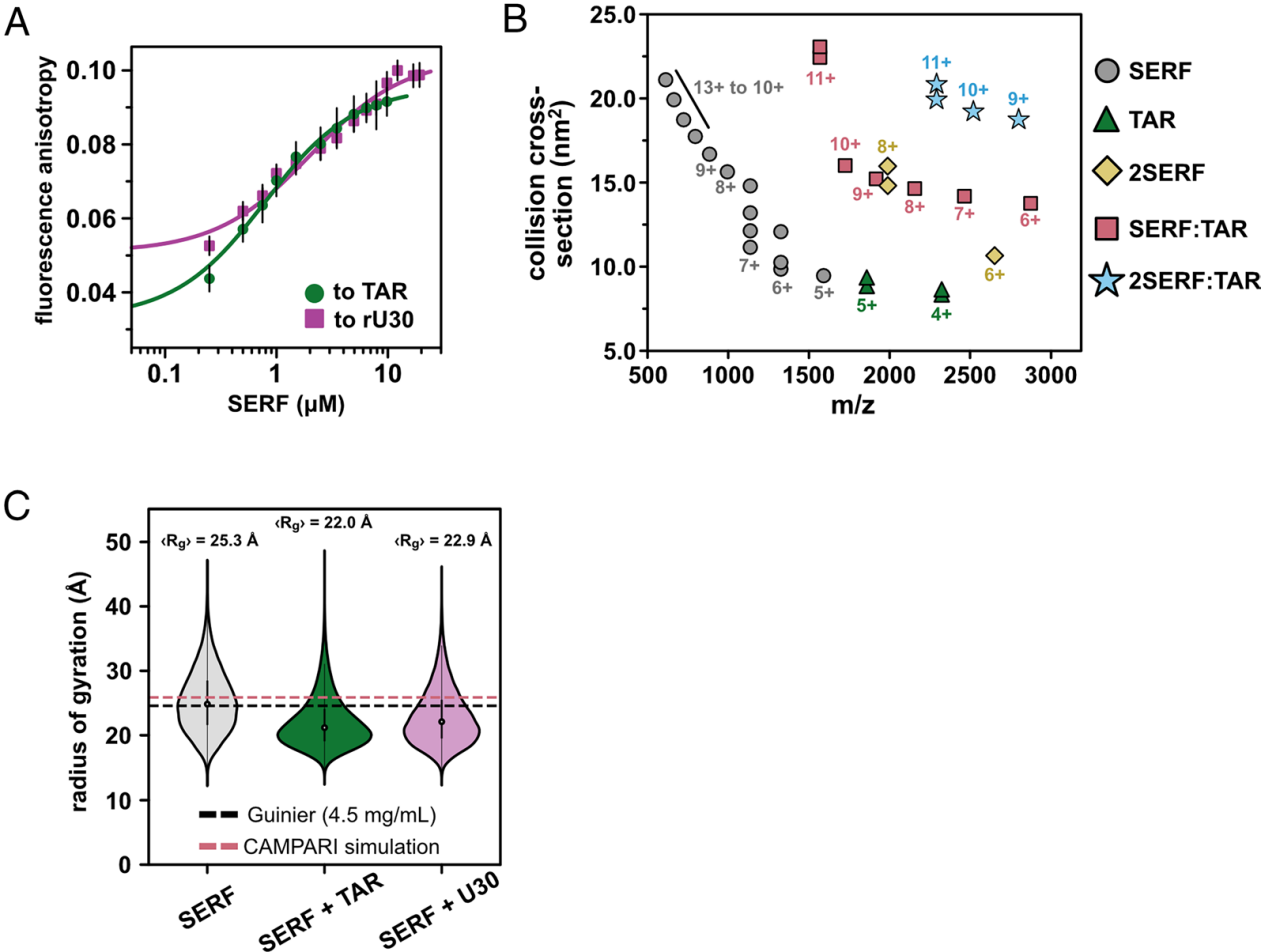
Properties of the SERF:TAR complex. (*A*) Binding isotherms for SERF and FAM-labeled TAR (circles) or FAM-rU30 (squares). The error bars represent the 95% CI from three technical replicates. The solid lines represent the nonlinear fit to a 1:1 binding model. The difference in baseline anisotropy values between binding TAR and rU30 arises from the difference in diffusion properties between the structured versus unstructured fluorescent RNAs. (*B*) Collision cross-section distributions (nm^2^) for the mixture of SERF and TAR determine by IM-MS. (*C*) Distributions of R_g_ values for SERF from CG simulations of SERF alone (*Left*), SERF and TAR (*Middle*), or SERF and rU30 (*Right*). Note that R_g_ calculations were performed on the protein molecule only. For each case, the average R_g_ is given above its corresponding violin distribution. For comparison, the average R_g_ from all-atom simulations is denoted by the dashed pink line. The R_g_ from Guinier analysis of the SAXS data is shown as the dashed black line.

Equipped with a detailed ensemble description of unbound SERF, we next examined the in vitro RNA binding behavior of SERF. An important feature of the SERF–RNA model system is the relatively high concentrations of SERF and RNA required to drive phase separation in the absence of PEG. This enables us to easily work in a concentration regime suitable for biophysical measurements while remaining below the threshold for phase separation. To describe the dilute-phase interactions of SERF with RNA, we measured SERF binding to the bulged stem loop of HIV-1 TAR (nucleotides 19 to 45, hereafter called “TAR”), by fluorescence anisotropy. We monitored the change in fluorescence anisotropy as a function of SERF concentration using fluorescently labeled RNA as the signal source (Fig. 3*A*).

By fitting these data to a 1:1 binding model, we determined that the SERF:TAR complex has an apparent dissociation constant (K_D,app_) of 0.67 ± 0.04 µM. Equilibrium binding measurements between SERF and fluorescently labeled (rU)_30_ suggest a modestly weaker interaction with this unstructured RNA (K_D,app_ = 1.9 ± 0.2 µM) as compared to the stem-loop TAR structure (*SI Appendix*, Table S3). Importantly, the charge stoichiometry for SERF (+12) and the RNA binding partners (−29 and −30) is not 1:1, so we also considered a model in which two SERF molecules could engage one RNA molecule (2:1) (*SI Appendix*, Fig. S3*A*). Ultimately, the 1:1 model was the most appropriate fit to the data, but we emphasize that the fitted constant is “K_D,app_” to account for potential “fuzzy” stoichiometry. The K_D,app_ values for SERF to both RNA partners are almost three orders of magnitude weaker than the binding affinity of TAR to its endogenous partner, the Tat protein (61).

To investigate potential conformational changes coupled to complex formation, we turned to native ion mobility mass spectrometry (IM-MS). Native IM-MS separates biomolecules based on their time of flight and net charge and can be used to determine global molecular dimensions (*Materials and Methods*). Native IM-MS and collision cross-section (CCS) measurements have been correlated previously with solution phase fluorescence measurements as well as the computed collision cross-section of published crystal structures suggesting that IM-MS can resolve changes in global conformations as small as 3% (62–64). For ions of the same mass, unfolded or structurally extended species typically have greater CCSs than folded or compact molecules of the same mass. The mass spectrum of an equimolar SERF and TAR mixture shows species corresponding to free SERF (monomer and dimer), free TAR, 1:1 SERF:TAR complex, and a 2:1 SERF:TAR complex (Fig. 3*B* and *SI Appendix*, Fig. S3 B and C).

Monomeric SERF yielded a broad charge-state distribution with charge states sampling 5+ to 13+, indicative of an extended conformational ensemble. As determined previously, the CCS distribution for SERF ranges from 9 to 22 nm^2^ (*SI Appendix*, Table S4) (65). SERF dimers were also detected in the IM-MS spectrum (65). It is possible that ionization in this method reduces intrachain electrostatic repulsion to a sufficient extent to permit SERF–SERF interactions, considering we see no evidence from other methods for SERF dimers. TAR ionizes in primarily two charge states (4+ and 5+) each with a bimodal CCS distribution. Together, across charge states, the IM-MS measurements of TAR reveal a narrow distribution of CCS populations, consistent with the conformational ensemble derived from NMR residual dipolar coupling data (66). The relatively narrow CCS distribution of the SERF:TAR complexes (from 14 to 22 nm^2^) compared to SERF monomers suggests a compaction of SERF associated with TAR binding.

To probe the structural determinants of SERF–RNA interactions in greater depth, we performed coarse-grained (CG) molecular dynamics simulations to model the SERF:RNA complex using the Mpipi force field (67). In this model, each amino acid or ribonucleotide is represented by a bead with residue type-specific size and interaction potential (68. In these simulations, SERF and (rU)_29_ RNA were modeled as flexible polymers, whereas TAR was modeled as a collection of TAR structures using the different models of the published TAR NMR structure (PDB ID: 1ANR) (see *Materials and Methods* for details). We simulated SERF alone or in a box with a structured TAR or unstructured (rU)_29_. For both RNA binding partners, SERF is indeed more compact (lower R_g_) in complex than in the unbound state (Fig. 3*C*), which is in line with our IM-MS results (Fig. 3*B*). We determined the relative affinity of SERF for these two RNA molecules from simulations (*SI Appendix*, Fig. S4 *A*–*D*) (3, 69, which revealed a ~3.5-fold higher affinity of SERF for TAR compared to (rU)_29_, in good agreement with the ~2.8-fold higher affinity seen by our experiments (*SI Appendix*, Table S3). Although there are inherent limitations to a CG model, such simulations provide a computationally tractable way to study the sequence-specific driving forces of protein–RNA complex formation.

### Flexible Regions Form the SERF–TAR Binding Interface.

**Fig. 4.**
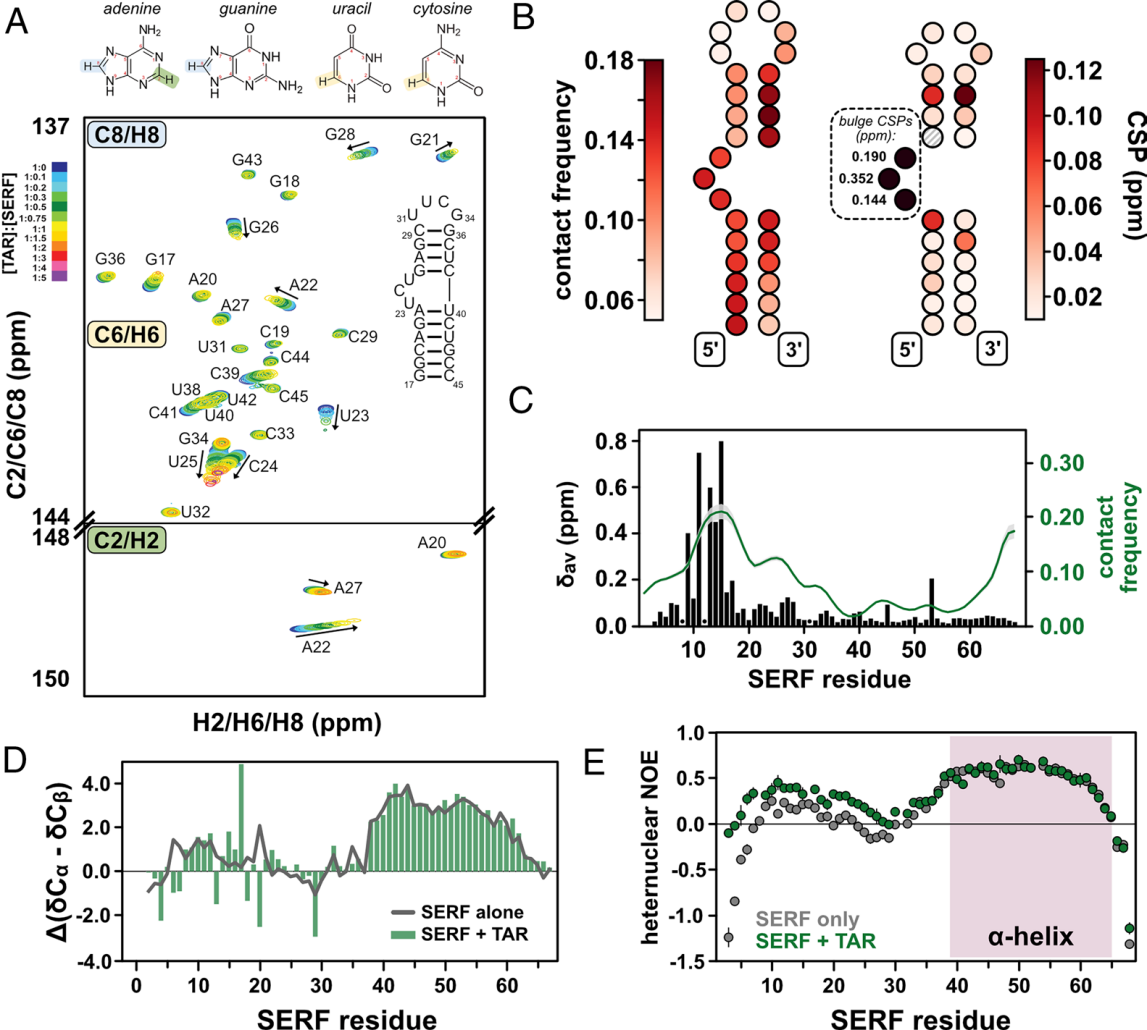
Determinants of SERF–TAR interactions. (*A*) TROSY-HSQC spectra of isotopically enriched TAR showing chemical shift changes upon titrating with unlabeled SERF (molar ratio color coded according to the insert scale). (*B*) Diagram of the binding footprint of SERF on TAR from CG simulations (*Left*) and NMR experiments (*Right*). Each TAR nucleotide is depicted with a circle colored to reflect either contact frequency (from simulations) or the maximal ^13^C CSP (from NMR). The intensity of the coloring represents the propensity for SERF interaction at each TAR nucleotide. The scale was adjusted to exclude the outlier bulge values in order to visualize other potential binding-induced CSPs. (*C*) Plot depicting regions of SERF that interact with TAR. CSPs in SERF upon adding TAR are shown as black bars (left axis). Residues for which bound-state assignments could not be determined are denoted with black dots. The average per-residue contact frequency for SERF with 15 TAR conformers is shown as a green trace; the SD is shown as gray shading above and below the green line. (*D*) Secondary structure profile for TAR-bound SERF calculated from NMR chemical shifts. The unbound SERF secondary structure profile is shown as a gray line for comparison. (*E*) {^1^H}−^15^N hetNOE profile for SERF in complex with TAR (green). Values for unbound SERF (gray) are reproduced from Fig. 2*B* for comparison.

To determine the nature of SERF–TAR interactions in the 1:1 complex, we again turned to NMR spectroscopy. In NMR experiments that detect signals from isotopically enriched TAR, we used a TAR mutant that contains a more stable tetraloop (UUCG) instead of the native hexanucleotide CUGGGA sequence (33). For NMR analysis, the tetraloop version of TAR was used due to the complex dynamics of the hexanucleotide loop sequence, which introduce challenges in spectroscopic interpretation (70). The uniform ^13^C/^15^N-labeling scheme for TAR allowed us to probe the chemical environment of nuclei in both sugar and nucleobase components. From a battery of 2D TROSY-HSQC NMR experiments (Fig. 4*A* and *SI Appendix*, Fig. S5 *A*–*D*), we observed large chemical shift perturbations (CSPs) in and around the TAR bulge (Fig. 4 *B*, *Inset*) upon the addition of SERF. SERF binding does not appear to alter the TAR secondary structure: Aside from fluctuations in the G28:C37 base pair of the upper helix, peaks for the canonical Watson-Crick base pairs of the TAR helices are maintained throughout the titration (*SI Appendix*, Fig. S5*A*).

The region of large CSPs in and around the UCU bulge due to SERF binding also functions as the recognition site for the HIV-1 Tat protein. However, despite binding to the same region, SERF shares no sequence similarity with the arginine-rich TAR-recognition motif in Tat (71). In addition to Tat, TAR has also been characterized for its interactions with the arginine analog argininamide (33). Tat binds TAR with ~nM affinity, whereas argininamide binds with ~mM affinity. Tat contains a specific Arg-rich motif that interacts with the TAR bulge, whereas SERF contains no such motifs (72). Although SERF lacks a bona fide TAR interaction motif, perhaps one of the nine Arg side chains can engage in a stacking interaction with the TAR bulge (as in the Tat protein) in a binding event that is primarily driven by electrostatic interactions (72). Despite their differences in affinity and binding chemistry, both Tat and argininamide can induce reorientation of the upper and lower TAR helices around the bulge (33, 71, 73). To determine whether SERF (~µM affinity) could induce a similar structural change, we performed residual dipolar coupling (RDC) experiments on isotopically enriched TAR with and without SERF.

Consistent with the observations for other TAR complexes, we found that SERF binding in a 1:1 stoichiometry decreases the TAR interhelical angle from a dynamic ensemble of conformations centered at ~52° (unbound) to more stacked conformations (bound) (*SI Appendix*, Fig. S5*E* and Table S5) (66, 74). Thus, despite differences in sequence and affinity compared to other partners, binding of SERF to TAR introduces an analogous structural change. Outside of the bulge nucleotides, the CSPs on TAR are fairly weak and well-distributed, suggesting that the SERF–TAR complex may be dynamic or fuzzy (Fig. 4*B* and *SI Appendix*, Fig. S6) (75). For comparison, we also quantified the contact frequency for each TAR bead in complex with SERF from CG simulations, which revealed a similar fuzzy binding “footprint” (Fig. 4 *A* and *B* and *SI Appendix*, Figs. S5 and S6). Together, these results suggest that the interaction between TAR and SERF is not mediated by SERF-bulge contacts alone, but that electrostatics may support contacts elsewhere on TAR toward formation of a nonspecific, fuzzy complex (33, 66, 71, 73, 74).

We next sought to identify the region(s) of SERF that supports its interaction with RNA. To do so, we added TAR to isotopically enriched SERF and compared the spectral features of SERF with or without RNA. We observed significant line broadening in the ^13^C, ^15^N-CON spectrum of SERF in complex, which posed a barrier to resonance assignments and interpretation of subsequent experiments (*SI Appendix*, Fig. S7 *A* and *B*). Therefore, we used ^1^H, ^15^N-HSQC experiments to monitor TAR-induced chemical shift differences in the SERF backbone. We unambiguously assigned cross peaks for 64 out of the 67 nonproline residues of SERF aided by the 2D ^13^C, ^15^N-CON, and 3D HNCO/HN(CA)CO spectra. The per-residue CSPs arising from the addition of TAR are primarily localized to the conserved NTR (Fig. 4*C*). Within this region, the CSPs near residues ~9 to 18 are up to 10 times larger than for the surrounding residues in the NTR. Notably, despite containing one of the three N-terminal Arg residues, this same region of SERF is somewhat devoid of CSPs upon binding to (rU)_30_ (*SI Appendix*, Fig. S7 *C* and *D*). Outside of this region, the CSP profiles for binding to TAR or (rU)_30_ are quantitatively similar and implicate the several N-terminal Lys residues as drivers of binding (Fig. 4*C* and *SI Appendix*, Fig. S7 *C* and *D*). From the PRE NMR experiments that probe the SERF NTR, the first ~20 residues appear to be locally compact (Fig. 2*C*). Therefore, we suggest that the large CSPs linked to TAR binding reflect decompaction and exposure of Arg side chains for interaction with the TAR bulge.

Interestingly, despite a high density of positive residues, the lack of CSPs in CTR suggests that this region does not interact directly with TAR (Fig. 4*C*, black bars). In support of this result, CG simulations of SERF and TAR show the highest contact frequency within NTR (Fig. 4*C*, green trace). Secondary structure calculations from the bound-state NMR measurements show that such features are largely unchanged from the unbound SERF ensemble (Fig. 4*D*), suggesting that the helical character in the CTR is maintained in the bound state. Taken together, the totality of data demonstrate that SERF shows no acquisition of structure upon binding to TAR. CG simulations of “SERF (1-34)” with TAR yielded the same relative dissociation constant as the full-length “SERF” with TAR’ (*SI Appendix*, Table S3), which supports the conclusion that the NTR is the primary interaction motif for these RNA partners.

Similar to our earlier experiments on unbound SERF, we measured ^15^N relaxation times to assess the dynamics of SERF in complex with TAR. A comparison of relaxation profiles of TAR-bound versus unbound SERF shows reduced backbone motions in SERF upon complex formation. The depressed *T*_2_ values of NTR in the TAR-bound versus unbound state may reflect slower conformational exchange in this region due to its interaction with the RNA (*SI Appendix*, Fig. S5*E*). hetNOE measurements of unbound and bound SERF also highlight the differences in flexibility between N- and CTR that arise from backbone structuring and/or RNA binding (Fig. 4*E*). Indeed, for TAR-bound SERF, the hetNOE in the NTR values are systematically larger than for unbound SERF and reflect the site of RNA interaction (Fig. 4*E*). Consistent with the secondary structure calculations from Cα and Cβ chemical shifts of TAR-bound SERF that suggest the CTR helix is unaffected by RNA interaction (Fig. 4*D*), the hetNOE profiles for the CTR are highly similar for the unbound and bound forms of SERF.

### SERF and TAR Exhibit Two-Phase Behavior at High Concentrations.

**Fig. 5.**
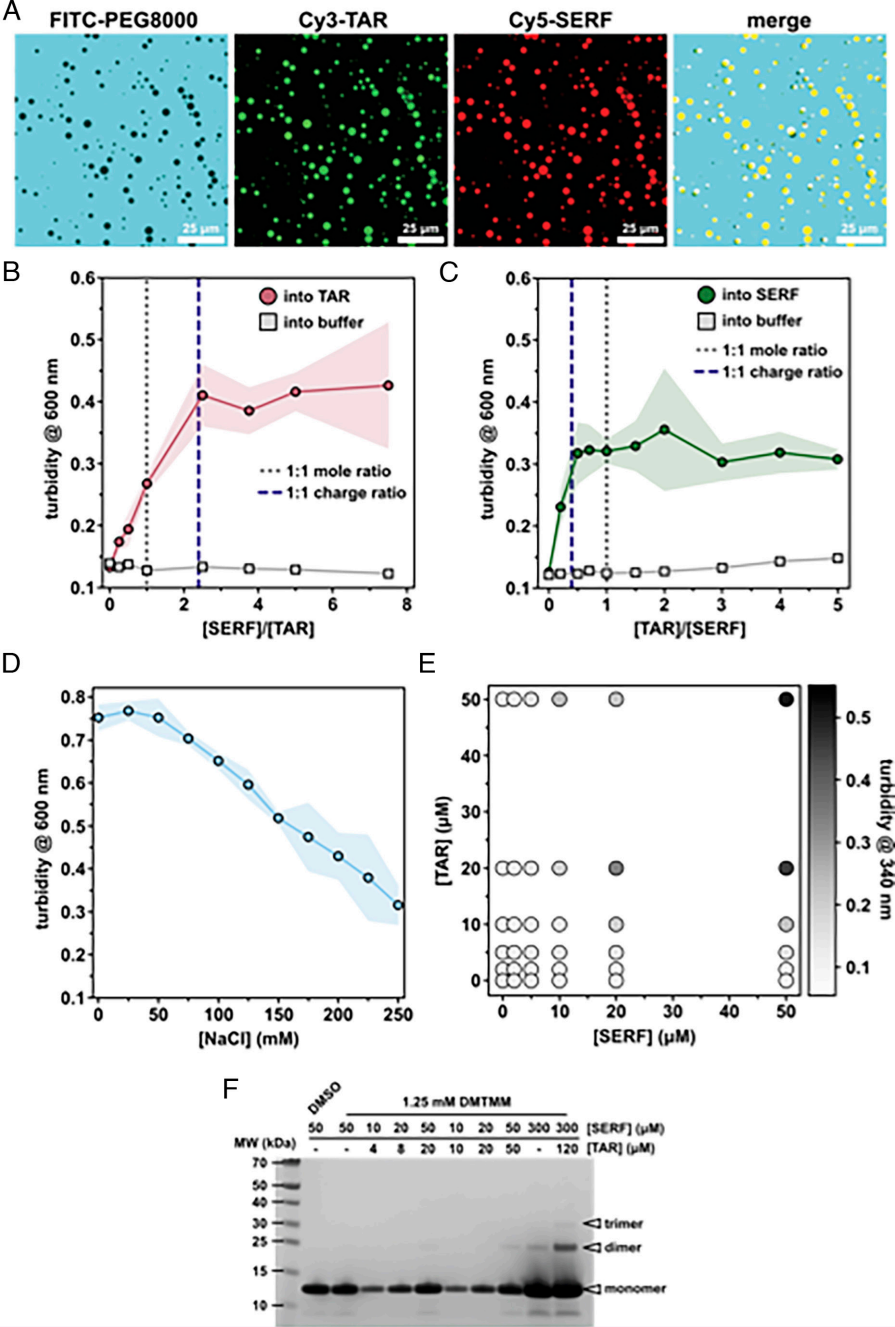
Characterization of SERF–TAR coacervates. (*A*) Fluorescence images of SERF-Cy5 (A63C) and/or TAR-Cy3 in droplets. Samples for imaging contained 50 μM SERF and 50 μM TAR and 10% (w/v) PEG8000 in a buffer of 20 mM HEPES, pH 7.5, 85 mM NaCl, and 1 mM MgCl_2_. FITC-PEG is not incorporated to droplets containing SERF and TAR. (*B*) and (*C*) Turbidity titrations (*B*) with increasing [SERF] titrated into a fixed concentration (20 μM) of TAR (pink circles) or buffer control (gray squares). (*C*) with increasing [TAR] titrated into a fixed concentration (50 μM) of SERF (green circles) or buffer control (gray squares). (*D*) Turbidity as a function of NaCl concentration. In (*B*–*D*) the shading represents the SD from the average of three technical replicates. Lines connecting the data points are shown to guide the eye. (*E*) Phase diagram of SERF–TAR phase separation generated by measuring turbidity at 340 nm. (*F*) SDS-PAGE gel showing DMTMM-crosslinked SERF species in the presence or absence of TAR RNA. Higher-order SERF species are observed only at high concentrations of SERF and TAR. The uncropped gel is found in *SI Appendix*, Fig. S8*C*.

Given the reported localization of SERF to the nucleolus, an RNA-rich membraneless compartment, we tested whether SERF could interact with TAR to form condensates in vitro (29). Indeed, Cy3-TAR and Cy5-SERF colocalize in solution into spherical, liquid-like droplets that can wet a microscope slide surface and can fuse and then relax into a larger droplet (Fig. 5*A* and *SI Appendix*, Fig. S8*A*). Although we observed droplet formation upon mixing SERF and TAR without a crowding agent (*SI Appendix*, Fig. S8*B*), the concentration required for phase separation of SERF–TAR mixtures is about 10 times lower in the presence of 10% (w/v) Polyethylene Glycol-8000 (PEG) (Fig. 5*E*). Under our experimental conditions, neither SERF nor TAR exhibited detectable phase separation behavior individually. Charge–charge repulsion, low hydrophobicity, and low aromatic amino acid content likely preclude homotypic interactions of SERF. Importantly, FITC-labeled PEG (average MW = 10 K) is excluded from the SERF–TAR droplets, which confirms that PEG is not cophase separating with SERF and TAR (Fig. 5*A*) (76).

We next investigated how the ratio of SERF to TAR influenced phase behavior. We used optical turbidity measurements (λ = 340 nm) to monitor phase separation of the SERF–TAR system at various molar ratios. Increasing SERF concentrations at a fixed concentration of TAR led to saturable increase in turbidity (Fig. 5*B*). We observed similar behavior in the reverse experiment, in which TAR was added to a fixed concentration of SERF (Fig. 5*C*). At pH 7.5, SERF has an estimated charge of +12, whereas the TAR net charge is estimated to be −29. The turbidity peaked at a stoichiometric ratio of approximately 2.5:1 for SERF:TAR, which is close to the charge-matched condition (Fig. 5*B*). Thus, in the SERF–TAR system, turbidity of the sample (indicating droplet formation) begins to plateau once a 1:1 charge ratio is achieved (Fig. 5 *B* and *C*) (22). In support of an electrostatically driven assembly mode, the phase separation of SERF and TAR is sensitive to ionic strength, as evidenced by a salt-dependent decrease in turbidity (Fig. 5*D* and *SI Appendix*, Fig. S8*D*). The charge complementarity between molecules and the salt dependence of their phase separation are hallmarks of complex coacervation, a type of associative phase separation that is driven by electrostatic attraction between oppositely charged polymers (77). The salt-sensitive coassembly and phase separation of SERF and TAR may be described by electrostatic driving forces alone; however, our observations do not eliminate the likely possibility that other interactions (i.e., cation–π) contribute to SERF–TAR phase behavior.

With a detailed description of the stoichiometric SERF:TAR complex and evidence for two-phase behavior at high concentrations, we sought more information about SERF–TAR assembly that precedes the emergence of a dense phase. To qualitatively assess the association of SERF monomers into higher-order species, we performed crosslinking experiments with a heterofunctional amine-to-carboxylic acid crosslinker, 4-(4,6-Dimethoxy-1,3,5-triazin-2-yl)-4-methylmorpholinium (DMTMM). DMTMM is a zero-length crosslinker that can couple acidic residues (Asp, Glu) with primary amines (usually Lys) (78). Following incubation with the crosslinker, the samples were assessed by Sodium dodecyl sulfate-polyacrylamide gel electrophoresis (SDS-PAGE) for higher-order SERF assemblies. DMTMM crosslinking experiments on a sample containing 300 μM SERF without RNA yielded a robust SERF monomer band and a very faint dimer band. In the crosslinking reaction containing 300 μM SERF and 120 μM TAR (1:1 charge ratio), we observed a more intense dimer band and evidence for a SERF trimer (Fig. 5*F* and *SI Appendix*, Fig. S8*C*). Although crosslinking approaches cannot distinguish between direct intermolecular contacts and spatial proximity of reactive groups on SERF in the presence of RNA, these results suggest that soluble SERF–TAR may explore higher-order assemblies in the background of a primarily binary complex.

## Discussion

In this study, we have investigated the in vitro interactions of a small, disordered protein, SERF, and a fragment of the HIV-1 TAR RNA and established this pair as a tractable model system for studying IDR interactions with RNA. SERF lacks a canonical, folded RNA-binding domain and instead leverages its high fraction of positive amino acids toward RNA binding. SERF also contains a transient α-helix in its CTR, but this region has no known functions or binding partners and does not appear to influence or be influenced by RNA binding. At high concentrations of SERF and RNA (or with the addition of a crowding agent), the pair undergo complex coacervation to form a protein- and RNA-rich dense phase. Importantly, the threshold for SERF–TAR phase separation is sufficiently high that we could use NMR spectroscopy to study the 1:1 complex to gain residue-level insights into this interaction. NMR measurements of SERF or TAR in complex suggest that neither molecule changes in secondary structure upon binding. Consistent with studies of TAR with other binding partners, we found that binding to SERF induces reorientation of stem loop helices around the TAR bulge (66). From IM-MS experiments and CG simulations, the SERF ensemble appears to modestly compact upon binding to RNA.

We find no evidence for higher-order assembly of SERF in the absence of RNA, which is likely a consequence of its charge and sequence features that make self-assembly electrostatically disfavored. From polymer theory, complex coacervation of oppositely charged polymers nominally consists of two thermodynamic steps: 1) entropically favored pairing between the positively and negatively charged polymers followed by 2) higher-order assembly into complex coacervates (79). Introducing a polyanion (RNA) effectively neutralizes the positive charges that preclude SERF–SERF interactions. This is captured by IM-MS experiments and CG simulations as a modest compaction of the SERF ensemble in the RNA-bound state. Mitigation of *intra*molecular repulsion by RNA binding should extend similarly to *inter*molecular repulsion between SERF molecules. This may ultimately support the emergence of SERF–SERF contacts that enhance the multivalency required for phase separation.

The electrostatic interactions and dynamics underlying complex formation and phase separation of two highly and oppositely charged disordered proteins were recently interrogated for the histone chaperone Prothymosin α and histone H1 (80, 81). At low concentrations, the pair form an extremely high-affinity (~picomolar) binary complex. With increasing concentrations, Prothymosin α and histone H1 undergo liquid–liquid phase separation and form salt-sensitive, viscous droplets that are stabilized by the same electrostatic interactions as those which stabilize the 1:1 complex (81). A recent investigation into RBP–RNA interactions within biomolecular condensates revealed that the sequence-specific RBP-RNA contacts that support dilute-phase interaction are also present in the dense phase (82). This study also reported the emergence of additional protein–RNA contacts in the dense phase arising, in part, from nonspecific, electrostatic interactions of RBP IDRs with the RNA phosphate backbone.

Whether this paradigm holds true for IDR–RNA interactions generally is unknown, but our finding that SERF compacts upon binding to RNA supports such a model for emergent contacts in higher-order assemblies via attenuated electrostatic repulsion. We note similarities between our system and a disordered protein that facilitates folding of its nucleic acid binding partner (4). This study reported accelerated folding kinetics of a DNA hairpin via nonspecific, electrostatic interactions with a highly positively charged disordered protein. In this system, the disordered protein acts as the counterion to permit nucleic acid compaction and base-pairing. Supporting the idea of charge screening as the primary factor in compaction, high-salt conditions recapitulated the compaction and increased rate of DNA folding (4). Although SERF does not fold upon binding RNA, the modest compaction of the ensemble implicates the same charge screening effect that promotes DNA folding upon binding to the polycationic protein.

For many RBPs, folded domains and intrinsically disordered regions jointly contribute to RNA-binding and phase-separation abilities (3, 68, 83). However, a consequence of high multivalency is that many such proteins readily phase separate, which poses a substantial barrier to characterize the residue-level details of their interactions (11, 25, 84). Proteome-wide studies to identify RBPs uncovered a remarkable fraction of binding sites that map to IDRs (85, 86). Thus, understanding the modes of interaction between IDRs and RNA will inform how RBPs with and without folded RNA-binding modules leverage IDRs in RNA recognition and binding. Through small IDR–RNA systems, like SERF–TAR, we may gain insights into the residue-level interactions that support a dilute-phase complex.

Recent studies of biomolecular condensate nucleation and early-stage assembly revealed that phase-separating proteins can form small “clusters” in the dilute phase that vary in the number of constituent molecules (87–89). The path from monomers to clusters to condensates is energetically complex, but the formation of relatively low-affinity clusters seems to be the first kinetic and energetic barrier to subsequent higher-order assembly (89). Although we lack evidence for cluster formation, the evidence of RNA-dependent SERF dimer and trimer may point to a similar mechanism at sufficiently high concentrations of each component. It is our hope that SERF and TAR [or unstructured (rU)_30_] can be a tractable system to enable further exploration of the general features of IDR–RNA interactions that support the various stages of condensate assembly.

## Materials and Methods

### Protein Expression and Purification.

The *S. cerevisiae YDL085C-A* gene that encodes the SERF protein was codon-optimized for expression in *Escherichia coli*. Point mutations were inserted into the SERF gene by site-directed mutagenesis with a QuikChange kit (Agilent) and were verified by Sanger DNA sequencing. The wildtype and mutant SERF genes were cloned into a pET28 vector that encodes an N-terminal His_6_-SUMO fusion tag for purification and scarless cleavage by ULP1 protease (65). Briefly, His_6_-SUMO-SERF was purified by Ni affinity chromatography, followed by treatment with the ULP1 protease (see *SI Appendix* for detailed purification methods) (90).

### Oligonucleotides.

Unless otherwise specified, the HIV-1 TAR RNA used for experiments has the sequence 5′-GGCAGAUCUGAGCCUGGGAGCUCUCUGCC-3′ (91). Unlabeled TAR RNA from Horizon Discovery and Cy3 3′-labeled TAR RNA from Integrated DNA Technologies were purchased as HPLC-purified lyophilized solids and were used without further purification. See *SI Appendix* for the preparation of lyophilized RNAs and for preparation of isotopically enriched TAR RNA.

### NMR Spectroscopy.

#### Chemical shift assignment and secondary structure analysis.

All NMR spectra were collected at 27 °C on a Bruker Avance NEO 600 (^1^H) MHz spectrometer with a 5 mm TCI triple-resonance cryoprobe. On-instrument processing of all spectra was performed using the Bruker Topspin software. Peak picking and manual assignments were carried out in NMRFAM-Sparky (92). Resonance assignments were acquired through ^13^C direct-detect spectra (40). Nearest-neighbor correlations between amide nitrogen atoms on adjacent residues were established using 3D (HACA)N(CA)CON and (HACA)N(CA)NCO experiments and 2D amino acid-filtered experiments collected as CACON/CANCO spectra for Asp, Ala, Glu, and CACON spectrum for Leu/Ala resonances (40, 93) Methods for additional NMR experiments and analyses are provided in *SI Appendix* (49).

#### SERF titrations and RDC measurements of TAR RNA.

All RNA NMR experiments were acquired with 0.5 mM ^13^C, ^15^N-enriched TAR, and varying concentrations of SERF in 15 mM Phosphate buffer (pH 6.4), 50 mM NaCl, 0.1 mM EDTA, and 10% D_2_O at 25 °C on Bruker spectrometer equipped with a QCI cryoprobe and operating at 600 MHz ^1^H frequency. Additional details about data collection and analysis are provided in *SI Appendix*.

### Small-Angle X-ray Scattering.

All SAXS data were collected at 20 °C. SERF samples for SAXS were buffer exchanged into 50 mM potassium phosphate (pH 6.5), 50 mM KCl, 1 mM MgCl_2_, 0.01% NaN_3_. Small angle X-ray scattering (BioSAXS) was collected on SERF (at 4.5 and 9 mg/mL), TAR RNA (at 3 and 5 mg/mL), and SERF–TAR complex at 250 µM. The concentration of SERF samples was determined using a Direct Detect Fourier-transformed infrared spectrometer (EMD Millipore). This was followed by reference buffer subtraction to get the raw SAXS curve from only the RNA. The data were analyzed using the ATSAS API (94). Additional details are provided in *SI Appendix*.

### All-Atom Simulations in CAMPARI.

All-atom simulations of SERF were performed using the ABSINTH implicit solvent model and the CAMPARI Monte Carlo (MC) simulation engine (https://campari.sourceforge.net/) using the parameter set *abs3.5_opls.prm*. ABSINTH has been used to generate experimentally validated ensembles of a range of IDRs (95). The SERF simulations presented consist of frames merged from at least six independent simulations; three simulations used a “random”/extended starting conformation and three used a “helical” starting conformation. Additional simulation details and analysis methods are provided in *SI Appendix* (43, 96–102).

### Fluorescence Anisotropy.

The apparent dissociation constant for the SERF–TAR complex was determined by titrating SERF into fluorescently labeled RNA and recording polarization change as a function of [SERF]. The data were fit assuming a 1:1 binding model using nonlinear minimization in SciPy and Python, which is described in detail in *SI Appendix*. Fitting of alternative models is also described in *SI Appendix* (103).

### Native IM-MS.

Lyophilized SERF and TAR were reconstituted in 100 mM ammonium acetate (pH 7.5) and mixed at a 1:1 ratio for 30 min prior to native ion mobility mass spectrometry analysis. See *SI Appendix* for additional information on sample preparation, data acquisition, and analysis (104–106)

### CG Simulations in LAMMPS.

CG molecular dynamics simulations of SERF with or without a given RNA molecule were performed using the LAMMPS simulation engine and the physics- driven Mpipi forcefield (85). In each case, the simulation box contained one molecule of “SERF” and one molecule of “RNA” under conditions optimized to observe binding/unbinding events See *SI Appendix* for additional details about simulation setup, analysis, and calculation of relative apparent dissociation constants (69).

### SERF–RNA Condensates.

Briefly, TAR was added to LLPS buffer [20 mM HEPES/NaOH (pH 7.5), 85 mM NaCl, 1 mM MgCl_2_] with 10% PEG 8000 (w/v), followed by SERF, to the desired mole ratio. The sample was mixed and maintained at room temperature until microscopy and turbidity measurements (described in *SI Appendix*). For in vitro cross-linking experiments, SERF and TAR mixtures in LLPS buffer were treated with the crosslinker DMTMM chloride and the resulting oligomers were visualized by SDS-PAGE (see additional details in *SI Appendix*).

## Supplementary Material

Appendix 01 (PDF)

## Data Availability

Some study data are available and have been deposited in various repositories. NMR data, which include standard Bruker files with raw time-domain data, pulse programs, shim data, acquisition and processing parameters, and sample information, along with UCSF Sparky format files and chemical shift assignments tabulated in Microsoft Excel spreadsheets, have been deposited in the Open Science Framework (OSF). Additionally, images from microscopy experiments in Leica Image Format (lif) files, mass spectrometry data, BioRad gel image files, and fluorescence and absorbance data in Microsoft Excel spreadsheets are also available in OSF. SAXS scattering profiles and associated data visualization files have been included as well. NMR assignments for SERF have been deposited in the BMRB database. Code and documentation related to data processing and simulations may be found at https://github.com/holehouse-lab/supportingdata/tree/master/2024/Mitra_Usher_etal_2024 (107). The source data for the figures and supplementary figures are included as a Source data file and have also been deposited in the Open Science Framework. The OSF can be accessed at https://osf.io/um368/ (108) for items 1-6, and the BMRB database can be accessed at https://bmrb.io/52619 (109) for item 7.
