# Supplementary material for: Molecular insights into the interaction between a disordered protein and a folded RNA
Source: Proc Natl Acad Sci U S A. 2024 Nov 26;121(49):e2409139121. doi: 10.1073/pnas.2409139121 (PMC11626198; doi:10.1073/pnas.2409139121)
Supplement: Supplementary file 1 — Appendix 01 (PDF) [file pnas.2409139121.sapp.pdf]

SUPPORTING INFORMATION FOR:

Molecular insights into the interaction between a disordered protein and a folded RNA

Rishav Mitra[a,b,1,2] ID, Emery T. Usher[c,d,1] ID, Selin Dedeoğlu[e] ID, Matthew J. Crotteau[a,b] ID, Olivia A. Fraser[f], Neela H. Yennawar[g] ID, Varun V. Gadkari[h,3], Brandon T. Ruotolo[h] ID, Alex S. Holehouse[c,d] ID, Loïc Salmon[e] ID, Scott A. Showalter[f,g] ID, and James C. A. Bardwell[a,b,4], jbardwel@umich.edu ID

[a]HHMI, University of Michigan, Ann Arbor, MI 48109
[b]Department of Molecular, Cellular, and Developmental Biology, University of Michigan, Ann Arbor, MI 48109
[c]Department of Biochemistry and Molecular Biophysics, Washington University School of Medicine, St. Louis, MO 63110
[d]Center for Biomolecular Condensates, Washington University in St. Louis, St. Louis, MO 63130
[e]Centre de Résonance Magnétique Nucléaire à Très Hauts Champs, UMR 5082, CNRS, Ecole Normale Supérieure de Lyon, Université Claude Bernard Lyon 1, Université de Lyon, Villeurbanne 69100, France
[f]Department of Biochemistry and Molecular Biology, The Pennsylvania State University, University Park, PA 16802
[g]The Huck Institutes of the Life Sciences, The Pennsylvania State University, University Park, PA 16802
[h]Department of Chemistry, University of Michigan, Ann Arbor, MI 48109
[i]Department of Chemistry, The Pennsylvania State University, University Park, PA 16802

[4]To whom correspondence may be addressed. Email: jbardwel@umich.edu.

Edited by H. Jane Dyson, Scripps Research Institute Department of Integrative Structural and Computational Biology, La Jolla, CA; received May 7, 2024; accepted October 16, 2024

Author contributions: R.M., E.T.U., S.D., N.H.Y., V.V.G., B.T.R., A.S.H., L.S., S.A.S., and J.C.A.B. designed research; R.M., E.T.U., S.D., M.J.C., O.A.F., N.H.Y., and V.V.G. performed research; R.M., E.T.U., S.D., N.H.Y., and V.V.G. analyzed data; J.C.A.B., B.T.R., A.S.H., L.S., and S.A.S. supervised the research; and R.M., E.T.U., S.D., and J.C.A.B. wrote the paper.

1 R.M. and E.T.U. contributed equally to this work.
2 Present address: Department of Bioengineering and Therapeutic Sciences, University of California San Francisco, San Francisco, CA 94158.
3 Present address: Department of Chemistry, University of Minnesota, Minneapolis, MN 55455.

This document includes:

Extended Materials and Methods
Figures S1 to S9
Tables S1 to S5

EXTENDED MATERIALS AND METHODS

Protein expression and purification

A gene fragment encoding the *S. cerevisiae* SERF protein (or point mutant) (Uniprot ID: *YDL085C-A*) was cloned into a pET28 vector with His_6-SUMO tags. The plasmid was transformed into *E. coli* BL21 (DE3) cells for protein expression. Cells were grown to early logarithmic phase at 37 °C in Protein Expression Medium (Gibco) or M9 minimal medium supplemented with $^{15}NH_4Cl$ (for expressing uniformly ^{15}N labeled protein), both containing 100 µg/mL kanamycin. Prior to induction, cells were cooled to 20 °C and then expression was induced by adding 0.1 mM isopropyl β-D-1-thiogalactopyranoside (IPTG). After 16 hours of protein expression at 20 °C, cells were harvested by centrifugation and resuspended in lysis buffer containing 40 mM Tris, 10 mM sodium phosphate (pH 8.0), 10% glycerol, three tablets of protease inhibitor cocktail (cOmplete mini EDTA-free, Roche), 0.375 mM $MgCl_2$, and 0.05 µg/ml each DNase I and RNase A. Cell pellets containing SERF were lysed by sonication on ice for 8 min, followed by lysate clarification via centrifugation twice at 37,500 ×g for 30 min at 4 °C. The clarified supernatant was loaded on a 5 mL HisTrap prepacked column (Cytiva) that had been equilibrated with lysis buffer. The column was washed with Lysis Buffer and the His_6SUMO tagged protein was eluted by adding Lysis Buffer containing 500 mM imidazole. To remove any undegraded nucleic acids, 500 units of Pierce™ Universal Nuclease (Cat# 88701) and 1 mM $MgCl_2$ were added to the eluate. ULP1 and 10 µL β-mercaptoethanol were also added to the eluted material to initiate SUMO tag removal. The resulting mixture was dialyzed against 40 mM Tris (pH 8.0), 300 mM NaCl overnight at 4 °C.

The cleaved affinity tag was separated from the SERF by passing the protein solution over a HisTrap column that had been equilibrated with Lysis Buffer. The flow-through fraction from the column containing the tag-free SERF was diluted by adding a cation exchange buffer (50 mM sodium phosphate (pH 6.0), 100 mM NaCl). Following centrifugation to remove insoluble material, the protein was passed over a HiTrap SP cation exchange column (Cytiva) that had been equilibrated with the cation exchange buffer. The protein was eluted with a linear gradient using a buffer containing 1 M NaCl. The eluted fractions were concentrated and loaded on a HiLoad Superdex S75 gel filtration column (Cytiva) equilibrated with 40 mM HEPES (pH 7.5), 100 mM NaCl. Uniformly ^{15}N and ^{13}C isotope- enriched proteins were expressed following the protocol of Marley et al. in M9 minimal medium supplemented with $^{15}NH_4Cl$, U-^{13}C-glucose, and ISOGRO growth supplement (Sigma).[1]

Purified SERF was flash-frozen in liquid nitrogen, and either aliquoted and stored at -80 °C, or subjected to overnight dialysis in 50 mM ammonium bicarbonate pH 8.0 at 4 °C followed by freeze-drying and storage at -80 °C. Protein concentration was determined with the Qubit protein assay (ThermoFisher Scientific) following manufacturer's protocol.

TAR RNA preparation

HPLC-purified TAR RNA was purchased as lyophilized powder. All solutions were prepared in nuclease-free water (Ambion) and buffers were prepared in DEPC-treated doubledistilled water to remove RNases. Typically, labeled RNA was dissolved in nuclease-free water to a stock concentration of 100 μM. The RNA was annealed by heating at 95 °C for 5 min followed by rapid cooling by plunging into ice. U30 RNA (Integrated DNA Technologies) was resuspended in 50 mM potassium phosphate (pH 6.5), 50 mM KCl, 1 mM $MgCl_2$, 0.01% NaN_3 buffer and used directly (i.e., no annealing protocol) for NMR experiments.

^{13}C-^{1}N-labeled TAR RNA preparation

The HIV-1 TAR containing a UUCG loop instead of the CUGGGA loop was produced by *in vitro* transcription with T7 RNA Polymerase High-Concentration (New England Biolabs) in 40 mM Tris HCl (pH 8), 1 mM Spermidine, 0.01% Triton X-100, 5 mM DTT, 28 mM $MgCl_2$, and 4mM ^{13}C,^{15}N-enriched NTPs (Eurisotop). The transcription reaction ran for 4 hours at 37°C on a heater/mixer block (Eppendorf ThermoMixer), in presence of 240 nM of semi-double stranded DNA template containing two 5' C2'-methoxy nucleotides. The reaction was quenched with 10% v/v 0.5 M EDTA. The purification was performed by ion-exchange high pressure liquid chromatography (HPLC) (Agilent) and using DNA-Pac PA1000 column at 80 °C (Thermo Scientific). The elution was performed by using a buffer containing 12.5 mM Tris (pH 8.0), 8 M urea, and a gradient of NaCl (from 0 to 0.5 mM) and detected by UV signal at 260 nm. Fractions were pooled together, butanol concentrated, and then precipitated by ethanol and sodium acetate overnight. The sample was washed with ethanol and remaining traces of ethanol were evaporated using a SpeedVac. The sample was resuspended in NMR Buffer (15 mM potassium phosphate buffer (pH 6.4), 50 mM NaCl, 0.1 mM EDTA, 10% D_2O), denatured by heating at 95 °C for 5 minutes, and cooled down at room temperature. The concentrations of RNA samples were measured by using NanoDrop 2000 spectrophotometer and adjusted to a final concentration of 0.5 mM.

NMR spectroscopy of SERF

Sample preparation

^{13}C, ^{15}N -SERF was buffer-exchanged into 50 mM potassium phosphate (pH 6.5), 50 mM KCl, 1 mM $MgCl_2$, 0.01% NaN_3 using a desalting column and concentrated to 0.8 -1 mM. Samples for NMR experiments were made to a volume of 500 μL with 5 % D_2O for the deuterium lock.

Secondary Structure Analysis

Side-chain chemical shifts were assigned by collecting 3D CCCON spectra for aliphatic carbon and 3D H(CC)CON aliphatic proton resonances[2]. The resonance assignments were mapped onto the ^{1}H,^{15}N-HSQC spectrum using standard 3D HNCACB, CBCA(CO)NH, HNCO, and HN(CA)CO spectra. Secondary structure propensity was determined by calculating residues specific secondary chemical shifts, i.e., the difference in the measured $^{13}C^{\alpha}$ and $^{13}C^{\beta}$ chemical shifts from the sequence-specific random coil chemical shifts calculated by the *ncIDP* predictor for the SERF amino acid sequence[3].

Spin Relaxation Measurements

^{1}N T_1 and T_2 spin relaxation data were collected as pseudo-3D spectra using $^{1}H_N$-start CON experiments developed previously for ^{13}C direct-detect spin relaxation measurements[4]. For T_1 measurements, 13 fully interleaved spectra were collected with relaxation delays of 20, 50, 80, 100, 150, 180, 200, 300, 400, 600, 750, 800, 1000, and 2000 ms. For T_2 measurements, 16 fully interleaved spectra were collected with relaxation delays of 15.68, 31.36, 62.72, 78.4, 94.08, 109.76, 125.44, 156.8, 172.48, 188.16, 219.52, 235.2, 250.88, 282.24, and 313.6 ms (the asterisks indicate duplicate measurements). The decay curves were fitted to a single exponential function (**Eq. 1**). Reported error bars represent uncertainty in fitting for each data point.

$$I = I_0 e^{\left(\frac{-t}{T_{1,2}}\right)} \qquad (1)$$

Spin-Labeling Cysteine Mutants

Because wild-type SERF lacks native cysteines, we expressed and purified variants containing single cysteines at position 63 (A63C) or 10 (A10C) for attachment of the paramagnetic nitroxide spin label, MTSSL (1-Oxyl-2,2,5,5-tetramethylpyrroline-3-methyl methanethiosulfonate). MTSSL stock was prepared in acetone at a concentration of 100 mg/mL. ^{13}C, ^{15}N -SERF A10C sample was diluted to 1 mL in Tris buffer, and freshly prepared DTT was added to a final concentration of 10 mM (>10-fold molar excess). The SERF samples were incubated for at least 1 hour at 4 °C in the dark to reduce cysteine. The samples were then buffer-exchanged into 50 mM potassium phosphate (pH 6.5), 50 mM KCl, 1 mM $MgCl_2$, 0.01% NaN_3 using a PD-10 buffer exchange resin (Cytiva) to remove the excess DTT. MTSSL was added to a final concentration of 1 mg/mL, and the sample was incubated for 1 hour at room temperature (~22°C) in the dark. The unconjugated MTSSL was removed from MTSSL-labeled SERF samples using a PD-10 resin equilibrated with 50 mM potassium phosphate (pH 6.5), 50 mM KCl, 1 mM $MgCl_2$, 0.01% NaN_3. Labeling was confirmed by mass spectrometry.

Paramagnetic Relaxation Enhancement (PRE) Measurements

A ^{1}H-start variation of the CON spectrum ((HACA)CON) was acquired on the freshly prepared paramagnetic sample containing spin-labeled A10C or A63C SERF in 50 mM potassium phosphate (pH 6.5), 50 mM KCl, 1 mM $MgCl_2$, 0.01% NaN_3 supplemented with 5% D_2O. Following data acquisition, the MTSSL radical was quenched by mixing 1 µL of 0.5 M sodium ascorbate directly into the NMR tube containing 500 µL of the sample. Following incubation at room temperature for 10 minutes, identical NMR spectra were acquired for the diamagnetic sample. PREs were reported as the ratio of cross-peak intensities under paramagnetic (I_{para}) and diamagnetic conditions (I_{dia}).

Protein NMR experiments on the SERF-TAR complex

The NMR samples contained 100 µM ^{13}C, ^{15}N-SERF and 100 µM TAR or 100 µM U30 in 50 mM potassium phosphate (pH 6.5), 50 mM KCl, 1 mM $MgCl_2$, 0.01% NaN_3 buffer supplemented with 8% D_2O. Resonance assignments of SERF in the presence of TAR were generated based on the traditional ^{1}H, ^{15}N HSQC experiment. The 3D experiments that were collected for assignments include HNCO, HN(CA)CO, CBCA(CO)NH, and HNCACB. Spectral comparison was carried out using the assigned HSQC spectrum of SERF alone as a reference. ^{15}N relaxation experiments on SERF in complex with RNA were conducted using 100 µM ^{15}NSERF and 100 µM TAR. The T_2 measurement was carried out with the same relaxation

delays as for SERF alone. The heteronuclear two-dimensional $\{^1H\} - ^{15}N$ nuclear Overhauser effect (hetNOE) was measured with a relaxation delay set to 5 seconds. The hetNOE profile was determined from the ratio of peak heights in the experiments with and without proton saturation.

TAR RNA NMR measurements

The titrations of SERF were observed through 2D ^{15}N- or ^{13}C-TROSY-HSQC spectra at concentrations of 0.05, 0.1, 0.15, 0.25, 0.375, 0.5, 0.75, 1, 1.5, 2, and 2.5 mM SERF. This range of concentrations resulted in TAR:SERF mole ratios spanning from 1:0.1 to 1:5. Combined chemical shift perturbation (CSP) (Δδ), were computed by:

$$\Delta\delta = \sqrt{\Delta\delta_H^2 + \left(\frac{\gamma_X}{\gamma_H}\Delta\delta_X\right)^2} \quad (2)$$

where γ_H and γ_X are the gyromagnetic ratio of ^1H and X (either ^{13}C or ^{15}N) and $\Delta\delta_H$ and $\Delta\delta_X$ the changes in chemical shift measured for the given nuclei (in ppm). Only peaks with a signal to noise ratio above 5 were kept in the analysis. Maximal ^{13}C CSPs correspond for each nucleotide to the maximal CSP observed for C1'H1', C2H2, C5H5, C6H6 or C8H8 in the 1:1 complex or to the latest observable fraction if the signal was unobservable at 1:1. The apparent dissociation constant of the interaction assuming a 1:1 complex was determined by non-linear fitting.

TAR RDCs (N1H1, N3H3, C1'H1', C2H2, C5H5, C6H6 and C8H8) were measured using pairs of 2D TROSY semi-anti-TROSY HSQC spectra recorded on a 1:1 TAR:SERF complex in presence of 25 mg/ml Phages (ASLA), obtained by mixing a 1 mM 1:1 complex with an equal amount of Phages (50 mg/ml in 15 mM phosphate buffer, pH 7.4). All spectra were processed using NMRPipe and analyzed in CcpNMR[5,6].

RDC data were analyzed using Module assuming idealized A-form helices for the two helices that were analyzed independently[5–7]. Scalar products between alignment tensors were computed as previously described[8]. It is worth noting that the RDCs were recorded on a 1:1 complex, which appear not to be saturated under NMR conditions and within a fast-intermediate exchange regime. Consequently, the obtained values do not report on the complex, but on a population weighted average of the free and bound form[9]. Therefore, only qualitative trends were extracted from this analysis. RDCs for the free TAR were taken from the literature[8].

Small-angle X-ray scattering

SAXS data were collected on freshly eluted samples from a Wyatt S-100 size-exclusion chromatography column using X-rays generated by a Rigaku MM007 rotating anode housed with the BioSAXS2000^{nano} Kratky camera system at a wavelength (λ) of 1.54 Å. The system includes OptiSAXS confocal max-flux optics that is designed specifically for SAXS and a sensitive HyPix-3000 Hybrid Photon Counting detector. The sample capillary-to-detector distance was 495.5 mm and was calibrated using silver behenate powder (The Gem Dugout, State College, PA). The useful momentum transfer scattering vector q-space range ($q = 4.\pi.\sin(\theta)/\lambda$, such that 2θ is the scattering angle) was generally from q_{min}= 0.008 Å^{-1} to q_{max}= 0.6 Å^{-1}. The energy of the X-ray beam was 1.2 keV, with the Kratky block attenuation of 22% and a beam diameter of ~100 μm. Protein samples were loaded using the Rigaku autosampler into a quartz capillary flow cell mounted on a sample stage cooled to 4°C and aligned in the X-ray

beam. The sample cell and full X-ray flight path, including beam stop, were kept *in vacuo* (< 1×10^{-3} torr) to eliminate air scatter. The Rigaku SAXSLAB software was programmed for automated data collection of each protein with elaborate cleaning cycles between samples. Data reduction including image integration and normalization, and background buffer data subtraction were also carried out using the SAXSLAB software. Six ten-minute images and three replicates from protein and buffer samples were collected and averaged after ensuring that no X-ray radiation damage had occurred. SAXS data overlays showed that there was no radiation decay or sample loss over the 60 minutes of data collection.

All-atom simulations in CAMPARI

For all-atom simulations, the *S. cerevisiae* SERF sequence (MARGNQRDLARQKNLKKQKDMAKNQKKSGDPKKRMESDAE ILRQKQAAADARREAEKLEKLKAEKTRR) with N- and C-terminal caps (acetyl and amide groups, respectively) was simulated in a spherical droplet with radius = 118 Å. The monovalent ion concentration (NaCl) was set at 0.05 M and was modeled explicitly. Each independent simulation reflects 50 million MC steps, of which the first 2.5 million were discarded as equilibration. The write-out frequency for accepted conformations was every 20,000 frames, such that the trajectories from each simulation contained >2,600 frames. Replicate trajectories from both starting conformations were merged to yield an ensemble of 14,250 total frames. Unless otherwise specified, all simulations were performed at 300 K; it is important to note that the simulation temperature does not necessarily reflect a true 300 K, but rather was chosen to allow sufficient sampling of conformational space. ABSINTH/OPLS-AA parameters were used over ABSINTH/CHARMM36 in order to prevent aberrant chain collapse.

For simulations of SERF with a fixed C-terminal helix, the AlphaFold2 (AF2)-predicted SERF structure was used to determine the boundary between N- and CTR of SERF. From the AF2 model, the C-terminal helix extends from residues 40 to 68, which is consistent with our experimental data showing high helical character beginning around residue ~38. A modified PDB file containing only the helix between residues 40 and 68 was used as the structured input for simulations of SERF with a fixed C-terminal helix. In simulations with the fully helical CTR, the backbone of residues 40-68 is constrained to an α-helix but the side chains were permitted to sample. Residues 1-39 were unconstrained and permitted to sample freely.

Simulation analyses

CAMPARI simulations were analyzed using MDTraj and SOURSOP using custom Python scripts (see Github)[10,11]. Ensemble distributions for the analytical Flory random coil (AFRC) model were generated using the AFRC Google Colab notebook (https://colab.research.google.com/drive/1WHw8ous7IgcKd2LKYuJLeBTlkdEYoRAk?usp=sharing). Paramagnetic relaxation enhancement (PRE) profiles were calculated from all-atom simulations as described in the references, but with modifications to account for the use of ^{13}C direct-detect CON experiments for these measurements[12,13]. Profiles were calculated for both spin label locations (residues 10 and 63); because the simulated ensembles do not contain the covalent spin label, PRE calculations were performed using distance measurements from Cβ of the corresponding residue in the native SERF sequence. For reproducibility, the protocol is described in detailed in a later section. SAXS profiles were calculated from all-atom simulations

using FoXS and then analyzed and visualized using BioXTAS RAW in the same manner as the experimental scattering data[14,15]. Structure visualization was performed using VMD or UCSF ChimeraX[16,17].

A comment on CTR helicity in all-atom simulations

Fig. 2A, which describes SERF secondary structure from NMR experiments and all-atom simulations, shows the failure of our simulations to capture the extent of the CTR helix observed in experiments. A close-up view of the per-residue average helicity (calculated by DSSP) highlights the location of the helical 'break', which centers on the residue Glu54 (**Fig. S1D**). Initially we suspected that this residue location and identity may be responsible for the helix break and performed a few additional simulations to this end. We simulated the following SERF sequences using coil starting conformations and five replicates each: wild-type (WT), E54A, E54D, E54Q, and $E54E_{protonated}$ ("E54X"). These were selected to survey potential effects from side chain charge, size, and chemistry.

The E54X variants have highly similar ensemble dimensions to the WT SERF, as described by radius of gyration (**Fig. S9A**). Compared to the fully helical simulation (constrained helix from residues 40 to 68), which has an ensemble-averaged Rg = 26.5 Å, a broken helix does not appear to appreciably contribute to ensemble dimensions. For each of the E54X variants, we also calculated average helical character using DSSP. There were no point mutations that 'rescued' the experimentally described helix; rather, each substitution decreased the overall CTR helical character compared to WT (**Fig. S9B**), suggesting that the trough of the helix break isn't solely the source of the problem.

Another possible explanation arises from the nature of the force field and solvent model (ABSINTH) employed in CAMPARI. Based on theoretical side chain pKa values, we generally assume that Lys and Arg will be protonated and that Asp and Glu will be deprotonated at pH 6.5 (NMR buffer pH). In experiments, charge regulation may support different charge states for these side chains, especially in a highly charged sequence. Charge regulation encompasses the phenomenon that the protonation state of an acidic or basic side chain may change depending on local sequence context (i.e., charge states of nearby residues) and solution conditions (i.e., buffer pH)[18–20]. However, surveying the combinatorics of the possible ionization states for all charged residues in SERF would make these simulations computationally intractable. The protonation states of all charged residues in SERF are fixed, which renders charge regulation impossible in the simulations. Hence, if such pKa shifting occurs in experimental conditions to support CTR helix stability, the simulations would not capture it.

Calculating PRE profiles from simulations

Profiles were calculated for both spin label locations (residues 10 and 63); because the simulated ensembles do not contain the covalent spin label, PRE calculations were performed using distance measurements from Cβ of the corresponding residue in the native SERF sequence. The ratio of cross-peak intensities between the paramagnetic (I_P) and diamagnetic (I_D) conditions is given by Equation 3:

$$\frac{I_P}{I_D} = \frac{R_2 \, exp\,[-R_2^{sp} t]}{R_2 + R_2^{sp}} \quad (3)$$

Where R_2 is the intrinsic transverse relaxation rate (set to 8.1 s^{-1} for carbon nuclei), and t is the total duration of the INEPT delays in the CON experiment (t = 50 ms). R_2^{sp} is the PRE arising from the spin label that is experienced by the carbonyl ^{13}C nucleus and is given by:

$$R_2^{sp} = \frac{K}{\langle r \rangle^6}\left(4\tau_c + \frac{3\tau_c}{1+ \omega_C^2 + \tau_c{}^2}\right) \quad (4)$$

Where $\langle r \rangle$ is the ensemble-averaged distance from the Cβ of the 'labeled' residue to the carbonyl carbon of every residue in our simulations. τ_6 is the rotational correlation time estimated from the SERF average T_1 and T_2 times to be ~2.5 ns[21]. ω_6 is the Larmor frequency of the excitation nucleus, which is ^{13}C (2π ×150.9 MHz) in CON experiments or ^1H (2π ×600 MHz) in (HACA)CON experiments conducted at field strength of 14.1 T. K is an atom-specific parameter given by:

$$K = \frac{1}{15}S(S+1)\gamma^2 g^2 \beta^2 \quad (5)$$

Where S is the spin angular momentum (S = ½ for ^1H and ^{13}C), g is the electronic g-factor (2.002319, unitless), β is the Bohr magneton (9.274009994 $\times 10^{-24}$ J/T), and γ is the gyromagnetic ratio of the start nucleus (10.705 MHz/T for ^{13}C or 42.576 MHz/T for ^1H). Hence, $K_C = 7.78 \times 10^{-34}$ cm^6/s^2 and $K_H = 1.23 \times 10^{-32}$ cm^6/s^2. ^{13}C direct-detect experiments for PRE used a modified ^{13}C, ^{15}N-CON pulse sequence that begins magnetization on H_α for improved sensitivity, so constants for ^1H were used in calculating PRE profiles from simulations. To generate the PRE profile null model assuming a Gaussian distribution of mean squared distances, $\langle r^\# \rangle$, the following from Meng et al. was used[13].

$$\langle r^2 \rangle = nl^2\left(\frac{1+\alpha}{1-\alpha} - \frac{2\alpha(1-\alpha^n)}{n(1-\alpha)^2}\right) \quad (6)$$

Where n is the number of residues between the site of the spin label and residue i, l is the monomer length (set to 3.8 Å), and α is the cosine of the bond-angle supplements given a freely rotating chain (set to 0.8).

Fluorescence anisotropy

The apparent dissociation constant for the SERF-TAR complex was determined by titrating SERF (0.5 mM stock) into a solution containing 15 mM sodium phosphate (pH 6.4), 50 mM NaCl, 0.1 mM EDTA, and 200 nM 3'6-FAM-labeled TAR RNA (100 µM stock) that was heated to 95 °C for 5 min and then cooled rapidly in an ice bath for 10 min or until use. Fluorescence signal was recorded at 25 °C with a Cary Eclipse Spectrofluorometer (Agilent) using excitation and emission wavelengths of 493 nm and 520 nm (5 nm bandpass for both) respectively, and anisotropy values were calculated using the following equations:

$$G = \frac{I_{hv}}{I_{hh}}\,;\, r_{obs} = \frac{I_{vv} - G.I_{vh}}{I_{vv} + 2G.I_{vh}} \quad (7)$$

where G is an instrument correction factor, I is the measured fluorescence intensity with polarizers oriented in directions that are indicated in subscripts (v is vertical and h is horizontal) and r_{obs} is the measured anisotropy. The data were fit assuming a 1:1 binding model using nonlinear minimization in using SciPy/Python.

The titration points could be fitted to a Langmuir-type isotherm describing the fraction of ligand (SERF) that is bound (FB):

$$FB = \frac{(K_D + [SERF]_{total} + [RNA]_{total}) - \sqrt{(K_D + [SERF]_{total} + [RNA]_{total})^2 + 4 \cdot [SERF]_{total} \cdot [RNA]_{total}}}{2 \cdot [RNA]_{total}} \quad (8)$$

where $[SERF]_{total}$ and $[RNA]_{total}$ are the concentrations of the ligand (SERF) and macromolecule (RNA), respectively, and K_D is the fitted dissociation constant (in the same units as the macromolecule and ligand concentrations). Fraction bound from **Eq. 8** relates to the spectroscopic observable (r_{obs}) through normalization based on the range of the measured anisotropy values:

$$r_{obs} = Q \cdot FB \cdot r_B + \frac{r_F(1 - FB)}{1 - FB(1 - Q)} \quad (9)$$

Where r_F is the anisotropy of the free fluorescent molecule, r_B is the anisotropy of the bound fluorescent molecule, and Q is a dimensionless correction value to account for differences in absolute fluorescence intensities between free and bound states. Using the measured r_{obs}, the fluorescent macromolecule (RNA) concentration (constant), and the total concentration of ligand (SERF) for each data point, we extracted K_D and Q by non-linear least squares fitting in Python.

To evaluate a binding model in which two SERF molecules can bind one RNA, we derived a partition function assuming two identical and independent binding sites (such that $K_{d1} = K_{d2}$ and no cooperativity). To make solving the two-site model tractable, we also assumed that $[SERF]_{total} = [SERF]_{free}$, although this assumption may not be reasonable in the concentration regime used for binding experiments[22]. Thus, we also compared to a 1:1 model making that same assumption. The 1:1 model with the simplifying assumption is uses **Eq. 8** to determine fraction of bound RNA (FB):

$$FB = \frac{\frac{[SERF]_{total}}{K_D}}{1 + \frac{[SERF]_{total}}{K_D}} \quad (10)$$

FB from **Eq. 10** was plugged into **Eq. 9** and the data were fitted as described above. The 2:1 model gives FB by the following partition function:

$$FB = \frac{\frac{[SERF]_{total}}{K_D} + \frac{[SERF]_{total}^2}{K_D^2}}{1 + \frac{[SERF]_{total}}{K_D} + \frac{[SERF]_{total}^2}{K_D^2}} \quad (11)$$

The FB from **Eq. 11** was similarly used with **Eq. 9** to fit for K_D and Q. In all cases, residual plots are shown to illustrate the quality of the fit (**Fig. S3A**).

Native IM-MS

TAR RNA was prepared by heating the RNA at 95°C for 5 min. After heating, magnesium acetate was spiked in at a final concentration of 2 mM, and the mixture was slowly cooled to 25°C for 30 minutes to allow the RNA to refold into a favored native-like structure. The RNA was exchanged into fresh 100 mM ammonium acetate (pH 7.5) to remove excess magnesium

acetate while retaining the Mg^{2+} ions bound to the RNA. Refolded TAR and SERF were mixed at a 1:1 stoichiometric ratio, with final concentrations of 4.5 μM each. The mixture was co-incubated for 30 minutes and analyzed by native ion mobility mass spectrometry.

For IM-MS experiments, buffer exchanges were performed using Micro Bio-spin size exclusion spin columns (Bio-Rad, Hercules, CA). Native ion mobility-mass spectrometry measurements were performed on a modified Agilent 6560 drift tube ion mobility quadrupole time-of-flight mass spectrometer optimized for native biomolecular measurements (Agilent Technologies, Santa Clara, CA)[23]. The instrument was operated in positive polarity, and under 99.999% nitrogen gas. Samples were introduced via nano- ElectroSpray ionization using 1300 kV of capillary voltage.

The instrument analysis settings were optimized to preserve native-like structure and non-covalent interactions. Briefly, the source desolvation gas temperature was operated at 20 °C, and gas flow was reduced to 1.5 L/min. The front funnel, trapping funnel, drift tube, and time-offlight tube were operated at 4.94, 3.80, 3.95, and 1.54×10^{-7} torr respectively. The drift tube was operated with an entrance voltage of 1700 V and an exit voltage of 250 V, enabling a low-field condition (18.125 V/cm). The IM arrival time distributions of ions were fit to gaussian functions, and the centroids of the fit gaussian functions were converted to rotationally averaged collision cross section ($^{DT}CCS_{N2}$) using the previously described single-field calibration using Agilent Tune Mix ions. IM measurements were performed in nitrogen, however for ease of comparison with previously published data, the CCS measurements were converted to $^{DT}CCS_{He}$ using a previously established relationship[24]. Both $^{DT}CCS_{N2}$ and $^{DT}CCS_{He}$ are reported in the supplemental information (**Table S4**). All data was analyzed using Agilent IM-MS Browser 10. Raw IM data extraction, and gaussian fitting were performed using CIUSuite2[25].

Setup and Analysis of Coarse-grained Simulations

Coarse-grained molecular dynamics simulations of SERF with or without a given RNA molecule were performed using the LAMMPS simulation engine and the physics- driven Mpipi forcefield[26]. In this model, each amino acid or nucleic acid monomer is represented as a single bead. Although we refer to the molecules in these simulations as 'protein' and 'RNA', it is more accurate to consider them as "protein- and RNA-flavored polymers". In Mpipi, lysine and arginine residues harbor relative charges of +1; aspartate, glutamate, and each nucleotide have relative charges of -1. Ionic strength is represented implicitly in Mpipi; for the simulations herein, the effective NaCl concentration was set to 0.05 M. This ionic strength allowed for the protein and RNA molecules to undergo several association and dissociation events over the course of the simulation.

Prior Mpipi simulations of the single-stranded RNA (ssRNA) homopolymer $(rU)_{40}$ showed excellent agreement with SAXS measurements of the same sequence[28]. Our simulations use $(rU)_{29}$ to be consistent with *in vitro* binding experiments and match the net charge of the TAR RNA. Unless otherwise specified, SERF and $(rU)_{29}$ are represented as flexible polymers and are permitted to sample physically relevant conformations. Importantly, we observe remarkable agreement in the SERF ensemble dimensions between SAXS experiments, all-atom (AA) simulations, and coarse-grained (CG) simulations of SERF alone ($R_g^{SAXS} = 24.9 \pm 0.1$, $R_g^{AA} = 25.9 \pm 0.8$, $R_g^{CG} = 25.4 \pm 0.1$).

Simulations of $(rU)_{29}$ ssRNA with SERF

Simulations were performed using a 40 nm^3 box with periodic boundaries. Unless stated otherwise, all simulation replicates were run for 400 million steps using a timestep of 10 fs. The first 0.25% (1 million) of the steps was discarded as equilibration steps and molecule coordinates were saved every 20,000 steps. Each system was simulated with ten independent replicates using the above settings; for SERF-$(rU)_{29}$, each replicate used a different random starting conformation to generate final trajectories of >199,000 total frames.

Simulations of CG 'TAR' RNA with SERF

Simulations containing structured TAR molecules were performed with the same simulation parameters but used a rigid CG representation of the TAR RNA instead of a flexible polymer. The Mpipi force field was initially developed to model biomolecular liquid-liquid phase separation and so is especially well-suited to study the SERF-RNA interactions. Given this specific utility, Mpipi was parameterized to model fully flexible polymers, not folded biomolecules. Therefore, to preserve its 3D structure, TAR was only allowed global rotational and translational moves (i.e., no sampling of conformational space at the residue/bead level). To account for this, we began with a published NMR structure of TAR (PDB ID: 1ANR) and used each of the 20 deposited conformers as a TAR structure for these simulations. Each conformer was simulated with flexible SERF in five independent replicates. Each replicate used the same rigid TAR conformer paired with a random starting conformation of SERF.

We converted the atomic coordinates of each TAR conformer PDB file into 'coarse-grained' format by removing atoms such that each nucleotide contained a single carbon atom (C1'). This is analogous to the use of Cα coordinates to generate a coarse-grain model of a folded protein domain from a PDB file[27,28]. It is important to acknowledge that, due to how nucleotides are represented in Mpipi, there is little difference between A, U, C, and G beads. And considering the previously demonstrated agreement in the behavior of rU40 in Mpipi and experiments, we opted to use only rU beads in the rigid representation of TAR[28]. This choice is supported by the remarkable consistency of SERF-TAR simulations with expectations based on SERF-$(rU)_{29}$ simulations and SERF-TAR experimental measurements (*discussed in next section*).

CG Simulation Processing and Analysis

Calculation of relative dissociation constants from simulations of 1:1 SERF: RNA complexes

Using the simulation parameters described above, we observed numerous binding and unbinding events between SERF and rU30 over the course of the simulation. The method used for determining an apparent K_D from CG protein-RNA simulations has been reported previously[28]. We first constructed a 'center-of-mass (COM) trajectory' in which each molecule is represented as a single bead whose position is given by the center of mass of the CG molecule at each frame. From each COM trajectory, we constructed a radial distribution function representing the intermolecular distances across all frames. The bimodal nature of this distribution arises from distance measurements between either two bound (short distance) or two unbound (long distance) molecules. The distribution was fitted with a two-Gaussian model. The threshold distance that defines whether the complex is 'bound' or 'unbound' was determined based on the intersection of the two Gaussians. Although the threshold distance nominally depends on the molecule sizes, the use of the same protein sequence and similar RNA lengths in different

simulations allows us to assume the same threshold distance across our analyses here. A threshold distance of 53.5 Å (determined from the SERF-$(rU)_{29}$ simulations) was used for all conformations of the SERF-TAR system. Frames were defined as 'bound' if the COM-COM distance was < 53.5 Å (**Fig. S4A**) and five or more consecutive frames fell below this threshold. The persistence of a complex for five or more frames was used as a proxy for the 'lifetime' that sets a true complex apart from a stochastic or random intermolecular encounter.

The fraction of assigned bound frames was used to calculate an apparent dissociation constant with analogy to the calculations from using the second Virial coefficient to account for the finite size of the simulation box[29–31]. The analyses presented from the CG simulations of SERF and TAR reflect the averages over 15 (of the original 20) conformers. To ensure the most rigorous analyses, we omitted simulations of TAR conformers that do not adequately sample the unbound state (**Fig. S4D**). All SERF-TAR conformer simulations were analyzed using the same pipeline (*see associated scripts on Github*); those for which SciPy did not fit an unbound-state Gaussian were omitted. Contact frequency is presented as a fraction from 0 to 1 based on the fraction of bound-state frames that contain residue pairs within 15 Å of each other. Smaller cutoff distances implicated the same residues in binding as those shown in **Fig. 4B-C**. Dissociation constants calculated across the 15 well-sampled pairs are between ~0.7 and 3.0 μM (based on normalization to SERF-$(rU)_{30}$ affinity from simulations and experiments), which is remarkably consistent with the enhancement of binding affinity between SERF-$(rU)_{30}$ and SERF-TAR measured in vitro (**Fig. S4C**).

Preparation of SERF-RNA condensates

SERF was reconstituted from lyophilized powder into the LLPS buffer (20 mM HEPES/NaOH (pH 7.5), 85 mM NaCl, 1 mM $MgCl_2$). TAR RNA was reconstituted in LLPS buffer and concentration was measured through measurements of absorbance at 260 nm in a Nanodrop ND-1000 spectrophotometer. Labeled TAR was reconstituted in RNase-free water and diluted into working solutions to the desired concentration. Protein concentrations were measured using Invitrogen Qubit Protein Assay kit IAW on a Qubit 2.0 fluorometer. Samples were prepared by sequentially combining LLPS buffer with 10 % PEG 8000 (w/v), TAR, and then SERF. Upon addition of SERF, 20% of sample volume was pipetted slowly for adequate mixing. All components were maintained at room temperature during sample preparation. For conditions in which phase separation was observed, sample turbidity increased concomitant with mixing indicating the rapid formation of two phases.

Microscopy

Condensate samples were added into Corning 96-well microplates with glass bottom (Cat#4580) pretreated overnight with 5% (w/v) Pluronic acid and rinsed thoroughly with ddH_2O. All fluorescent imaging were performed on a Leica SP8 confocal microscope equipped with LAS X Life Science Microscope Software and 100X objective lens. Phase-separated droplets contained 1 % labeled components. 3'Cy3-labeled RNA was imaged using 554 nm excitation and 568 nm emission. Cy5 labeled SERF A63C mutant was imaged using 651 nm excitation and 670 nm emission. mPEG-FITC, MW 10 K (Creative PEGWorks Cat# PSB-2253-100 mg) was imaged using 495 nm excitation and 519 nm emission.

Turbidity measurements

Turbidity measurements were taken at either fixed 20 µM of TAR with varying SERF concentrations and 50 µM of SERF with varying TAR concentrations. 50 µL of sample were added to wells in a 384- well, transparent, non-binding microplate from Greiner Bio-One (REF: 781901). Turbidity (light scattering) at room temperature was monitored as absorbance at 340 nm or 600 nm wavelengths using a Tecan M1000 infinite microplate reader. The effect of varying ionic strength was examined by adding small volumes of 5 M NaCl prepared in DEPC-treated ddH$_2$O water to 50 µL solutions containing 50 µM of SERF and 50 µM of TAR in LLPS buffer.

In vitro crosslinking

50 mM 4-(4,6-Dimethoxy-1,3,5-triazin-2-yl)-4-methylmorpholinium Chloride (DMTMM) stock solution was freshly prepared by dissolving 13.84 mg of DMTMM powder in 540 µL of anhydrous dimethyl sulfoxide. DMTMM solution was added to a final concentration of 1.25 mM to samples containing different concentrations of SERF and TAR in LLPS buffer (20 mM HEPES/NaOH (pH 7.5), 85 mM NaCl, 1 mM MgCl$_2$). The crosslinking reaction was allowed to proceed for 1 hour at room temperature. Excess unreacted DMTMM was quenched by addition of 1 M Tris pH 7.5 solution to a final concentration of 100 mM Tris. The quenching reaction was incubated for 45 min at room temperature. The residual crosslinker was removed by centrifugation and 100 µL supernatants were mixed with 25 µL of 5X reducing SDS-sample buffer. The samples were boiled at 95 °C for 10 min and run on a NuPAGE 4-12% Bis-Tris polyacrylamide gel (Thermo Fisher Cat#NP0322BOX). Bands were visualized by staining the gel with Pierce GelCode blue stain reagent following manufacturer’s protocol.

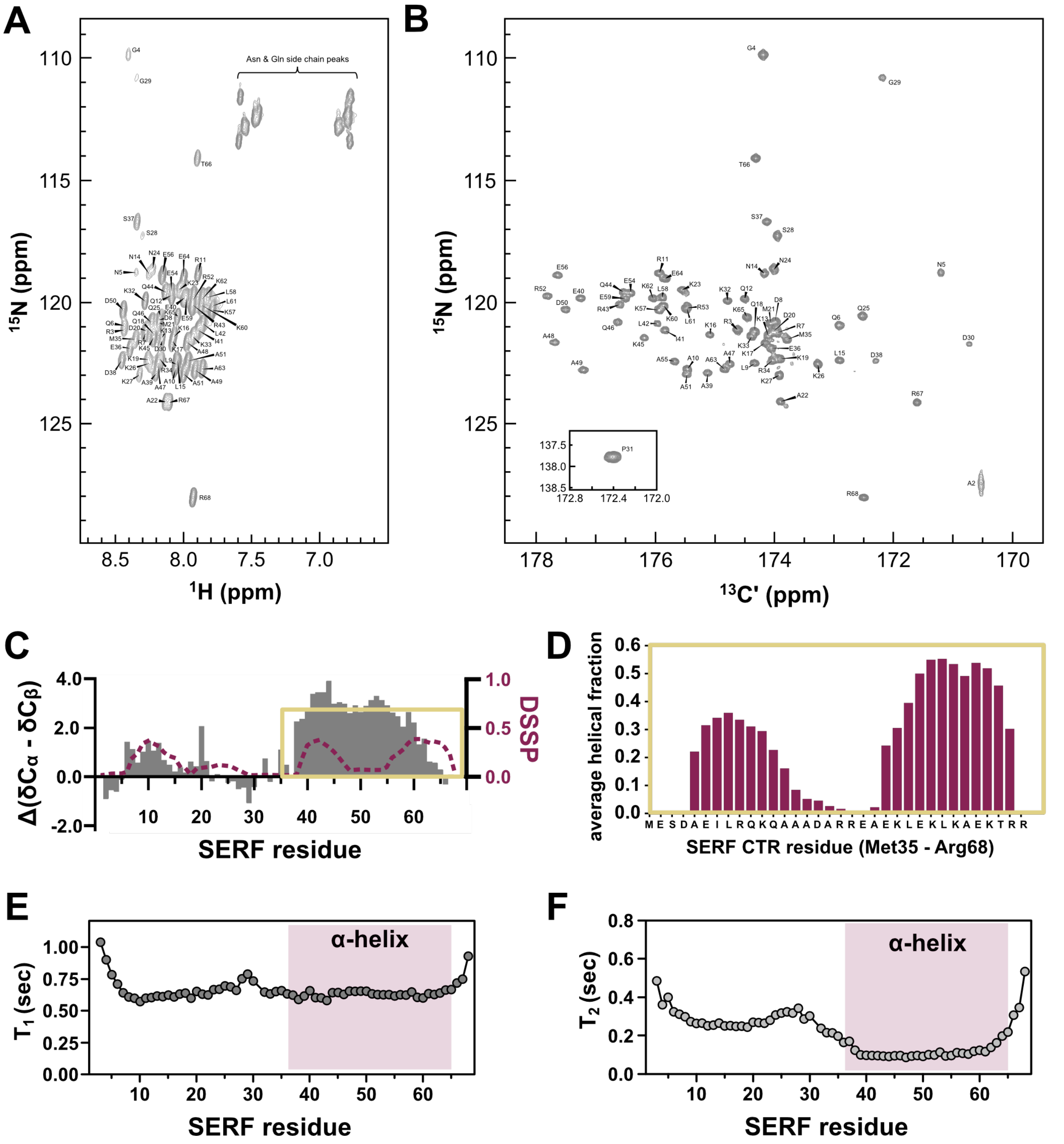

Figure S1: Local structural features of the SERF ensemble from NMR spectroscopy and all-atom simulations. (A) ^{1}H, ^{15}N-HSQC spectrum with SERF assignments (see also *Table S1*). **(B)** ^{13}C, ^{15}N-CON spectrum of SERF with assignments (see also *Table S1*). **(C)** Plot of SERF secondary structure from side chain chemical shifts (positive values indicate α-helix; negative values indicate β-strand) (left axis). The DSSP scores for α-helical character averaged over all simulation frames are shown as a dashed line (right axis). The yellow rectangle shows the C-SERF helical region of interest. **(D)** Zoomed-in representation of DSSP scores for CSERF helix from simulations. The breakpoint in the helix is centered around a stretch of charged residues (DARREAEK). **(E)** Plot of T_1 relaxation times per residue of SERF from ^{15}N spin relaxation. **(F)** Plot of T_2 relaxation times per residue of SERF from ^{15}N spin relaxation.

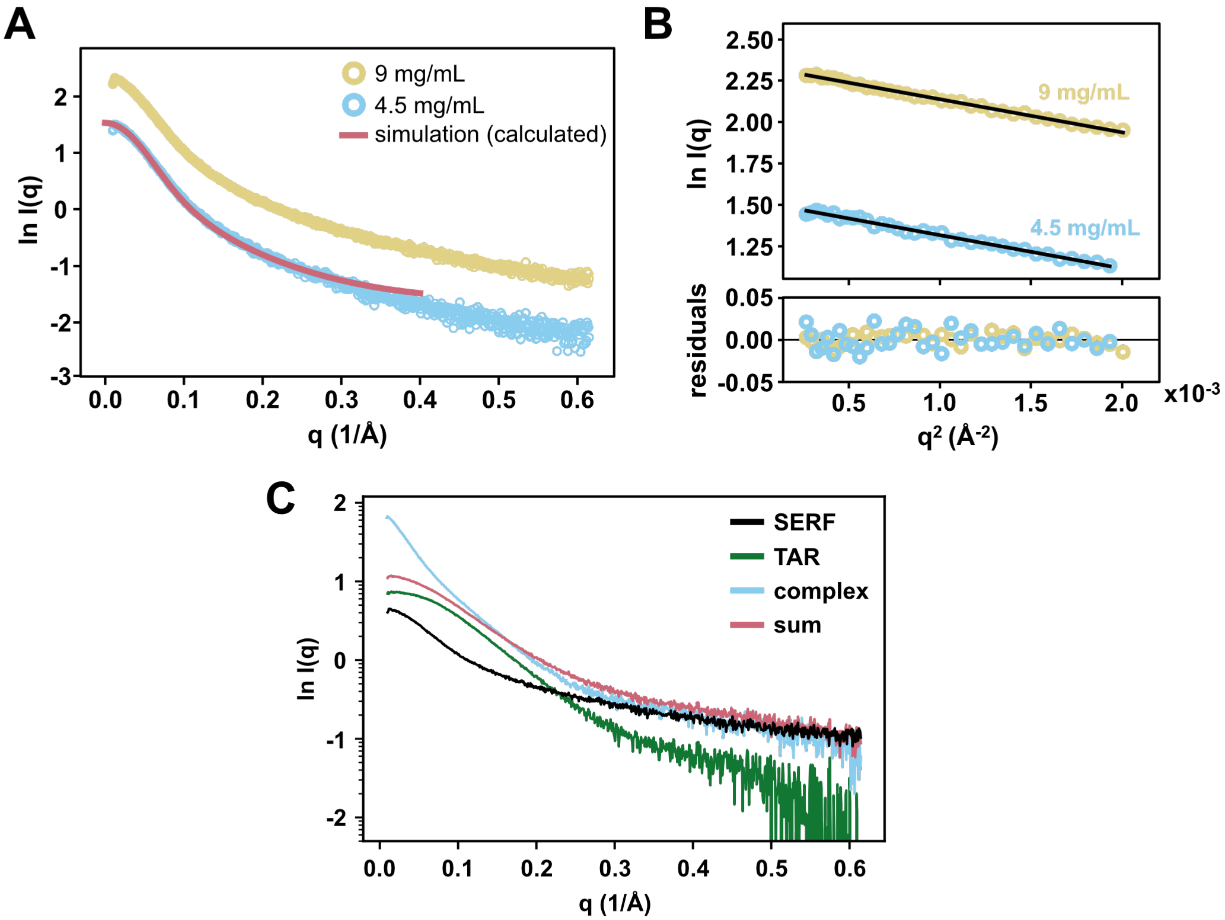

Figure S2: Global SERF ensemble characterization by SAXS. (A) Raw scattering curves for two concentrations of SERF shown with scaled SAXS profile calculated from all-atom simulations using FoXS[36]. **(B)** Guinier transformation of SAXS data at two concentrations with linear fit to approximate radius of gyration. The small spread of residuals suggests adequate linear fitting in both cases. **(C)** Raw scattering curves for each SERF (black) and TAR (green) alone, the sum of their scattering profiles (pink), and the scattering curve measured for the complex (blue).

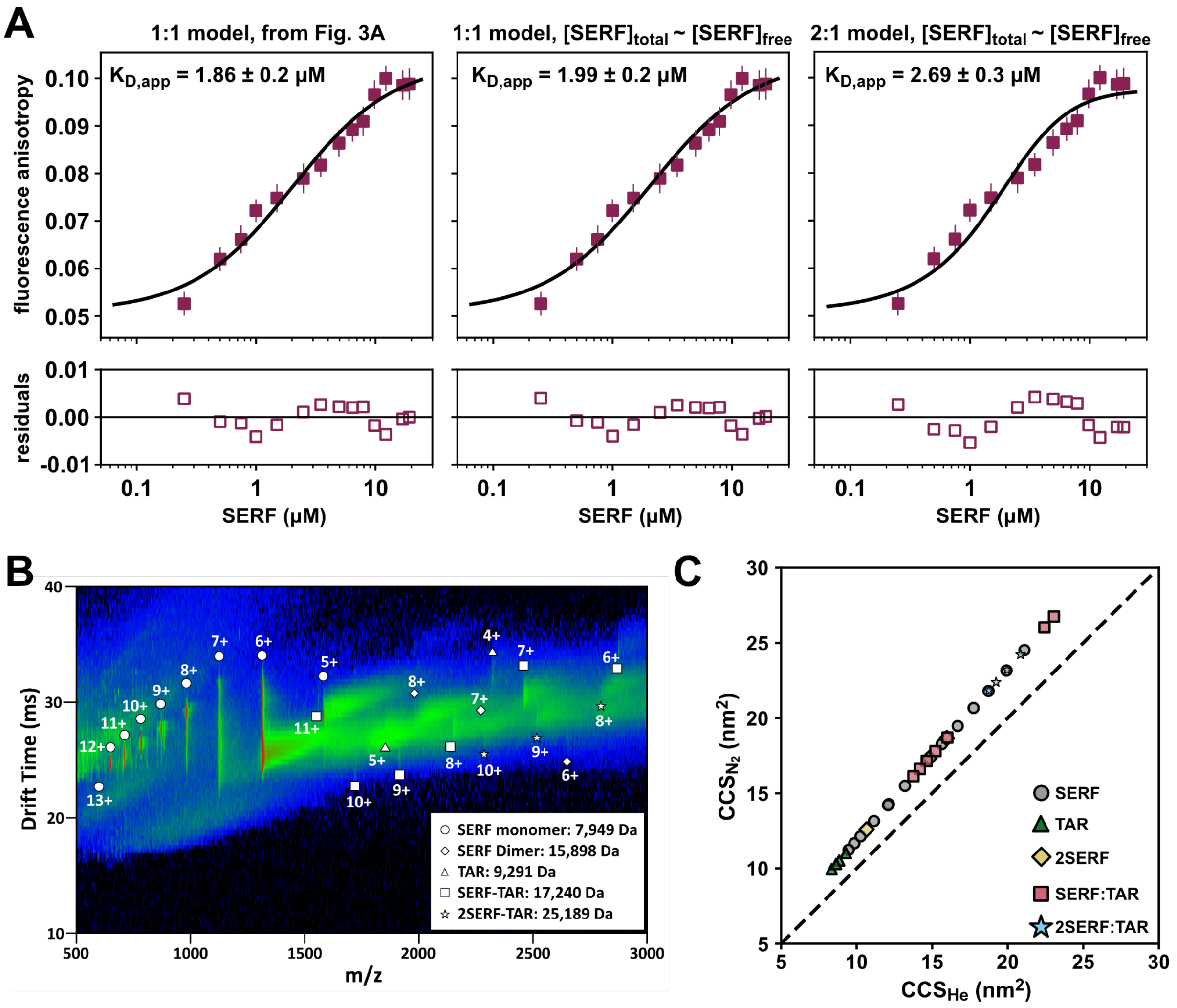

Figure S3: Characteristics of SERF-TAR complexes. (A) Comparison of fitting models for fluorescence anisotropy data. **(B)** Plot of drift time as a function mass/charge ratio used to determine collision cross-section distributions. Each feature is assigned with the biomolecule(s) and ionization state it represents using the shapes given in the legend. The molecular weights for different species are given in the legend. **(C)** Plot of collision cross sections from different carrier gases. The dashed line is the function y = x to guide the eye.

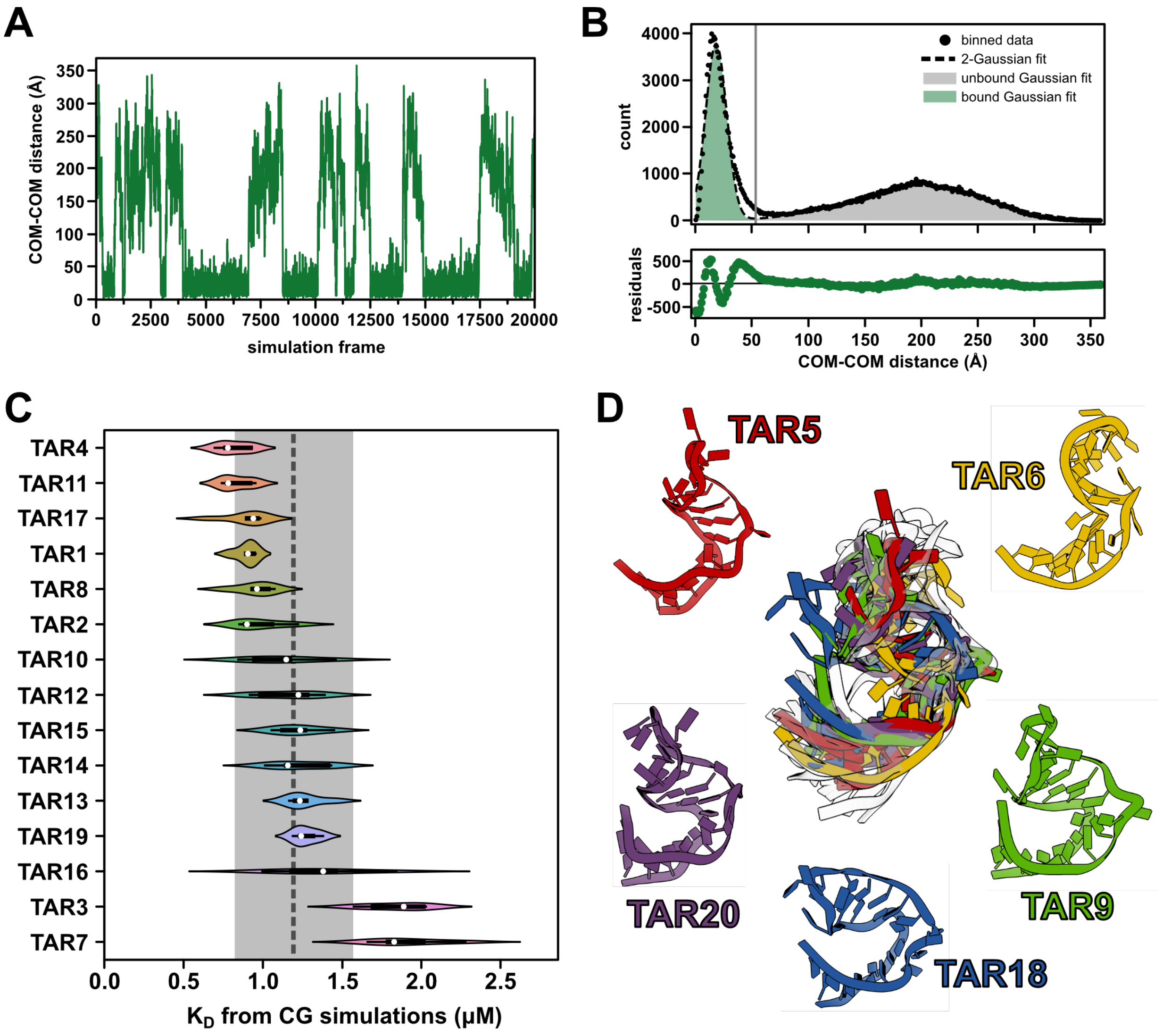

Figure S4: Monitoring 'SERF'-'TAR' binding by coarse-grained simulations using Mpipi. **(A)** Trace of distances between each 'SERF' and 'TAR' over the course of the simulation measured from the center of mass (COM) of each molecule. **(B)** COM-COM distances from **(A)** presented as a distribution and fit with a 2-Gaussian model. The green (low COM-COM distance) and grey (high COM-COM distance) shading reflects 'bound' and 'unbound' frame assignments, respectively. The grey vertical line at 52.5 Å is the 'cutoff' distance (see *Methods*) that minimizes the overlap of the two sub-distributions. **(C)** Distribution of K_D values across five replicates for each included TAR conformation ranked by average affinity. The vertical grey dashed line and shaded region represent the average and standard deviation of dissociation constants over all listed conformers. **(D)** Cartoon depiction of all 20 TAR conformations from PDB ID 1ANR aligned for visualization. Five colored TAR conformations that were omitted from average K_D calculations are shown in the overlay and separately.

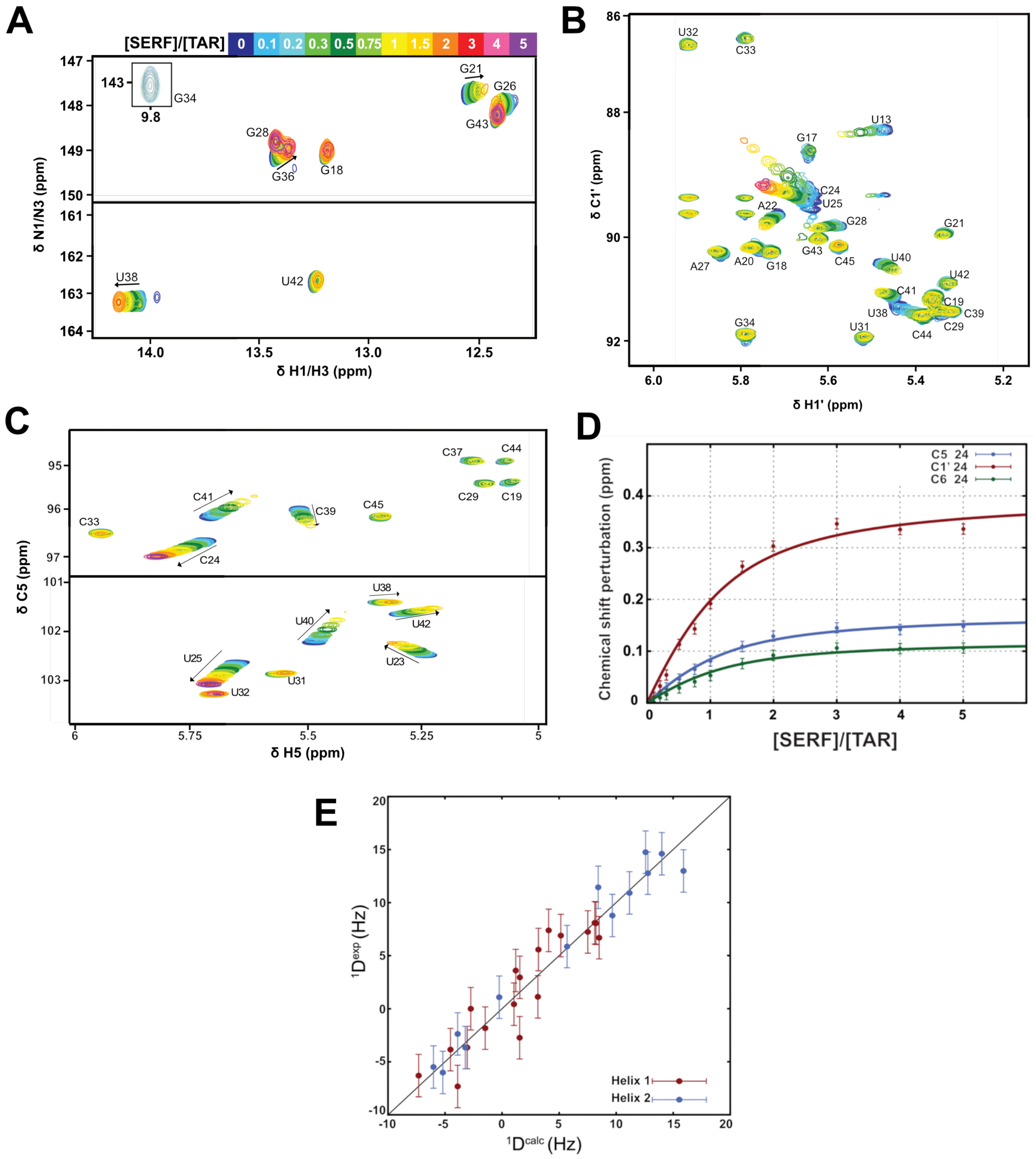

Figure S5: NMR spectroscopy of SERF-TAR interaction. (A) Imino, **(B)** C1', and **(C)** C5 TROSY-HSQC spectra of isotopically enriched TAR showing chemical shift changes upon titrating with unlabeled SERF (molar ratio color coded according to the scale in (A)). **(D)** Chemical shift perturbations (in ppm) of the residue C24 of TAR with increasing [SERF]/[TAR] from C5H5 (red), C6H6 (green), and C1'H1' (blue) spectra. The lines correspond to fits of the chemical shift perturbations of C1', C5 and C6 nuclei of C24 to a 1:1 binding model. **(E)** RDC reproduction of TAR assuming two independent ideal A-form helices. The associated alignment tensors are given in **Table S5**.

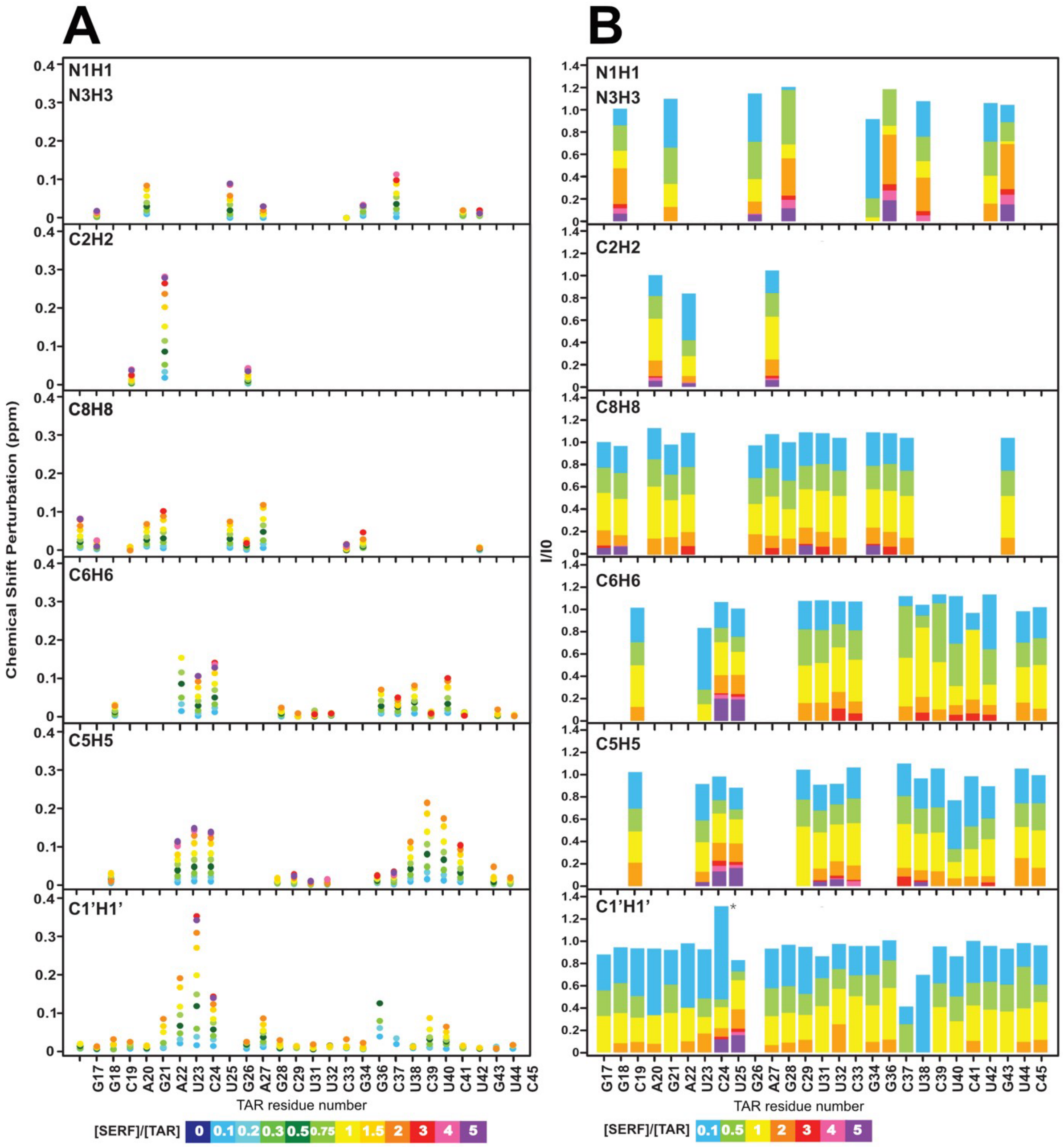

Figure S6: NMR titrations to probe the SERF-TAR interface. (**A**) Chemical shift perturbations and **(B)** intensity ratios of TAR for N1H1, N3H3, C2H2, C6H6, C8H8, C5H5, and C1'H1' spin pairs along the titration (**Fig. 4 and S6**). Molar ratio color coded according to the insert scale. The star (*) indicates a higher uncertainty for signal highly overlapping in the TAR alone spectrum.

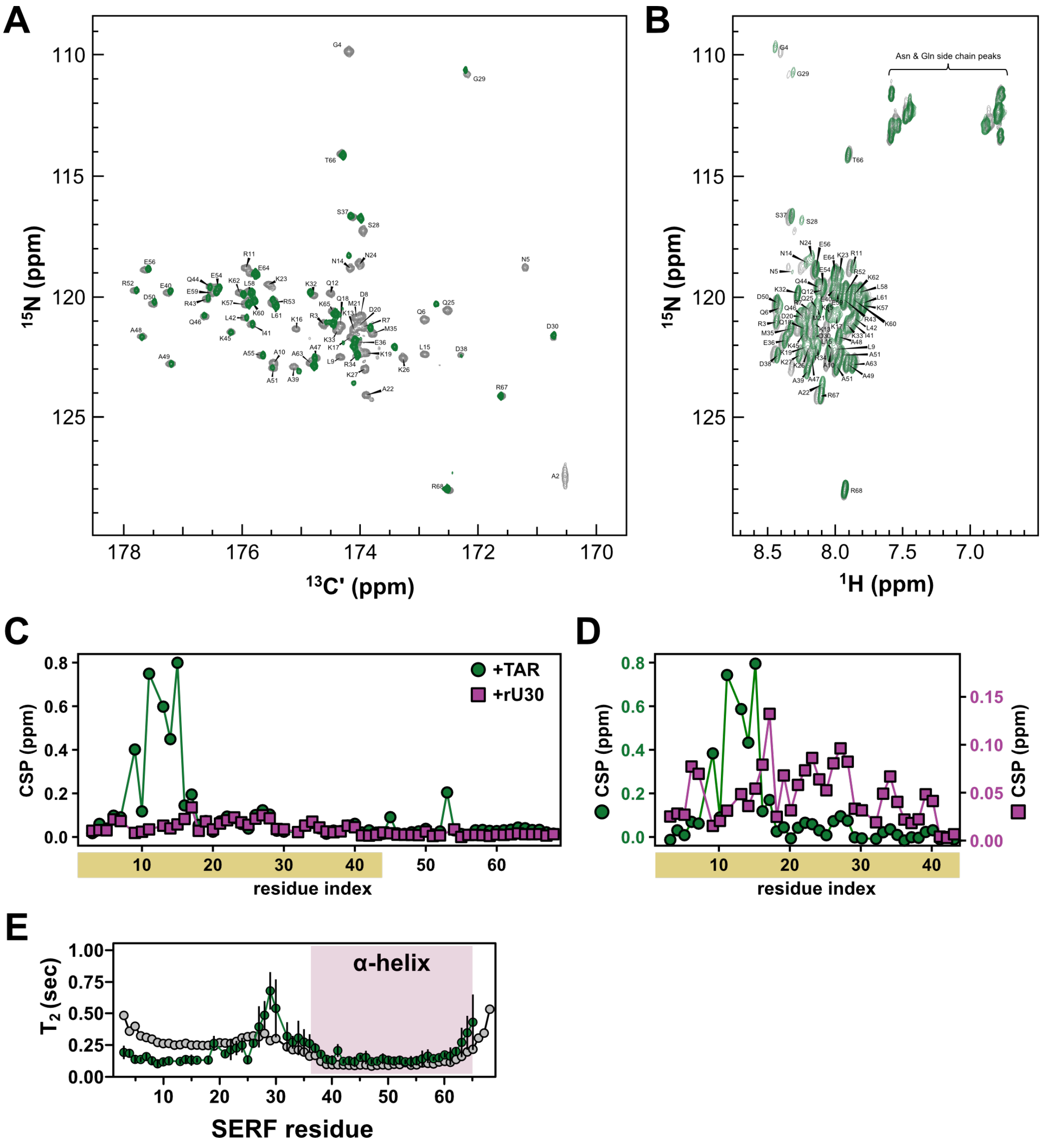

Figure S7: NMR spectroscopy of SERF bound to TAR RNA. (A) ^{13}C, ^{15}N-CON spectra of unbound SERF (grey, from *Fig. S2*) and TAR-bound SERF (green). Significant linebroadening of resonances for residues that directly contact RNA precluded use of the CON for analysis of chemical shift perturbations upon RNA binding. Resonance assignments are shown for unbound SERF. **(B)** ^{1}H, ^{15}N-HSQC spectra of SERF alone (grey) and bound to TAR (green). The peak assignments shown are those for the bound state, which were used to generate the plots in **(C)** and **(D)**. **(C)** Plot of chemical shift perturbations (CSP) on SERF for binding to TAR RNA (green circles) or rU30 (magenta squares) on the same axis set. The yellow highlighting along the x-axis denotes the region of SERF that is examined more closely in **(D)**. **(D)** Re-scaled representation of CSP plot from **(C)** to reflect the differences in CSP magnitude for the different RNA binding partners. **(E)** Plot of T_2 ^{15}N relaxation times for SERF alone (gray) and bound to RNA (green).

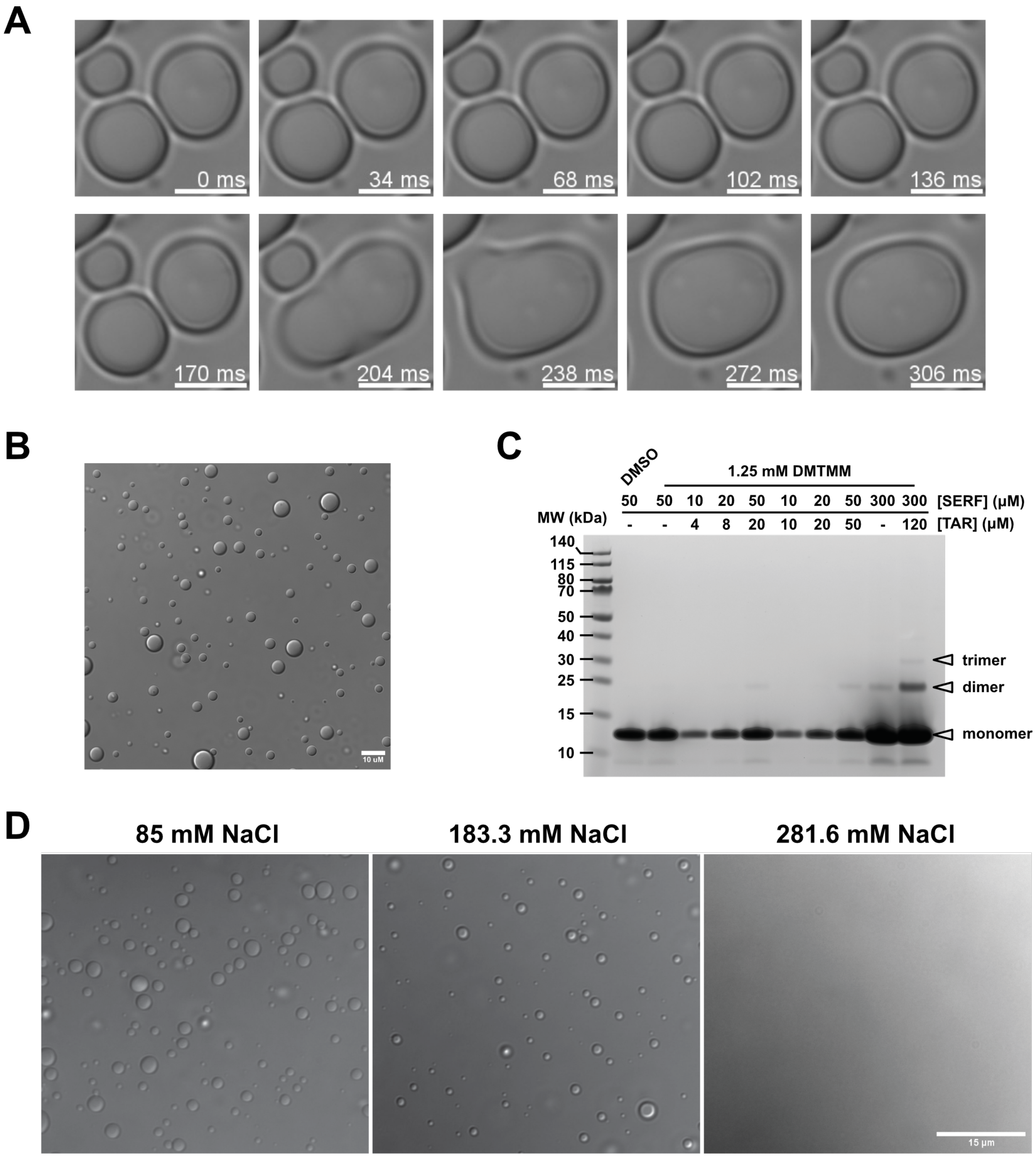

Figure S8: SERF and TAR undergo concentration-dependent assembly and phase separation. (A) Time-resolved DIC images of SERF-TAR droplets (in 10% PEG buffer) demonstrate their liquid-like behavior via droplet fusion. **(B)** DIC image of SERF-TAR droplets composed of 125 μM TAR and 312.5 μM SERF in 0% PEG. **(C)** Uncropped image of SDS-PAGE gel showing DMTMM-crosslinked SERF species in the presence or absence of TAR RNA. **(D)** DIC images of SERF-TAR droplets (in 10% PEG buffer) showing sensitivity to increasing NaCl concentrations.

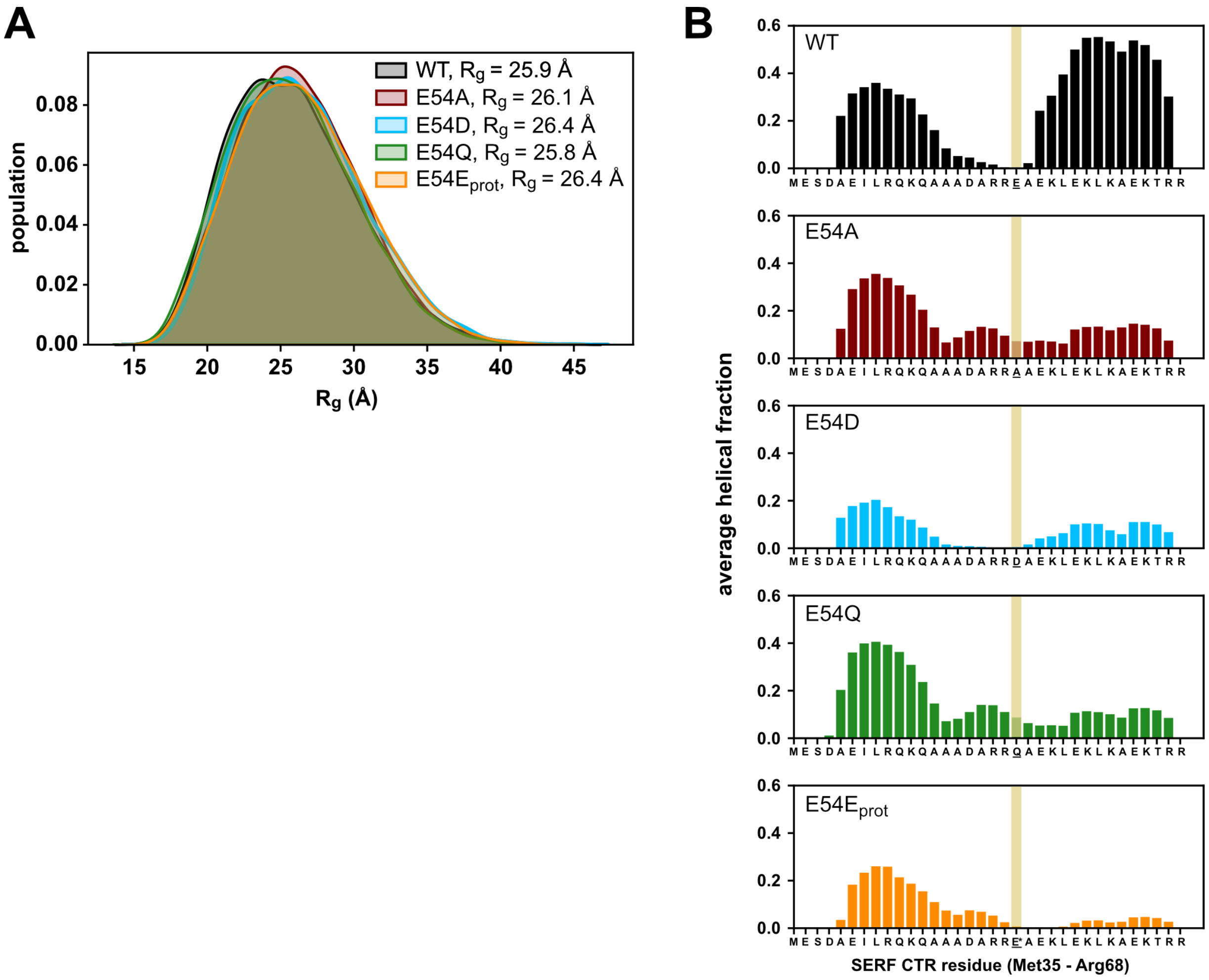

Figure S9: SERF CTR helicity as described by all-atom simulations. (A) Radius of gyration distributions presented as kernel density estimates for each E54 variant of SERF. The ensemble-averaged Rg is given in the legend. **(B)** DSSP plots showing ensemble-averaged helical fraction for each variant of SERF at residue 54. The underlined amino acid is that of position 54 and the yellow vertical is presented at the same position to guide the eye.

Table S1: Chemical shift assignments for SERF from ^{13}C direct-detect NMR experiments. Atoms with missing assignments denoted by –'-' are those for which we do not expect to observe a resonance (i.e., we cannot detect the amide nitrogen for N-terminal residue in ^{13}C-detected experiments; and Gly residues do not have Cβ atoms).

Residue #	Amino acid	Cα (ppm)	Cβ (ppm)	CO (ppm)	N (ppm)
1	M	52.26	30.65	170.5	unk
2	A	48.96	16.47	174.6	127.5
3	R	53.37	28.27	174.2	121.1
4	G	42.63	-	171.2	109.9
5	N	50.35	36.16	172.9	118.8
6	Q	54.12	26.2	174.0	121
7	R	54.28	27.7	174.0	121.3
8	D	51.97	38.18	174.3	120.8
9	L	53.39	39.14	175.5	122.5
10	A	50.74	15.83	175.9	122.7
11	R	54.25	27.66	174.5	118.8
12	Q	53.93	26.33	174.1	119.9
13	K	54.51	30.17	174.2	121.4
14	N	50.9	35.86	172.9	118.8
15	L	53.03	39.48	175.1	122.4
16	K	54.04	30.11	174.2	121.4
17	K	54	30.18	174.3	121.7
18	Q	53.63	26.59	173.9	121.2
19	K	54.29	30.35	174.0	122.3
20	D	51.89	38.27	174.1	121.2
21	M	53.15	29.3	173.9	120.9
22	A	49.66	16.17	175.5	124.1
23	K	53.96	30.18	174.0	119.5
24	N	50.64	35.97	172.5	118.6
25	Q	53.2	26.73	173.3	120.6
26	K	53.62	30.22	173.9	122.5
27	K	53.38	30.36	174.0	123
28	S	55.67	61.16	172.2	117.3
29	G	42.2	-	170.7	110.8
30	D	49.43	38.54	172.4	121.7
31	P	60.91	29.5	174.8	137.8
32	K	54.04	29.79	174.4	119.9

Residue #	Amino acid	Cα (ppm)	Cβ (ppm)	CO (ppm)	N (ppm)
33	K	53.48	30.22	174.0	121.4
34	R	53.75	27.87	173.8	122.4
35	M	53.21	29.39	174.0	121.5
36	E	54.32	27.46	174.1	121.9
37	S	55.85	61.26	172.3	116.7
38	D	53.38	37.76	175.1	122.4
39	A	52.01	15.79	177.2	122.9
40	E	55.74	26.43	175.8	119.8
41	I	61.23	34.96	176.0	121.2
42	L	54.95	38.79	176.6	120.9
43	R	56.33	27.49	176.5	120.1
44	Q	55.72	25.54	176.2	119.6
45	K	56.42	29.78	176.6	121.5
46	Q	55.16	25.71	174.7	120.8
47	A	51.65	15.23	177.7	122.5
48	A	51.52	15.32	177.2	121.7
49	A	51.9	15.32	177.5	122.8
50	D	53.54	37.68	175.5	120.3
51	A	51.82	15.22	177.8	123
52	R	55.64	29.74	175.5	119.8
53	R	56.15	27.49	176.4	120.3
54	E	55.85	26.45	175.7	119.6
55	A	51.99	15.38	177.6	122.4
56	E	55.92	26.77	175.9	118.9
57	K	56.07	29.73	175.9	120.3
58	L	54.27	39.01	176.5	119.8
59	E	55.88	26.66	175.9	119.9
60	K	55.48	27.11	175.5	120.1
61	L	53.85	39.36	176.0	120.2
62	K	55.08	30.01	174.8	119.8
63	A	50.29	16.11	175.8	122.8
64	E	54.44	27.23	174.5	119
65	K	53.98	30.15	174.3	120.6
66	T	59.44	67.67	171.6	114.1
67	R	53.39	27.98	172.5	124.1

Table S2: SERF ensemble dimensions as described by radius of gyration.

system	method	R_G (Å)	error (type)
sequence-based predictions			
SERF	AFRC (random coil)	20.8	N/A
	ALBATROSS	24.8	N/A
X-ray scattering (experiments)			
SERF	Guinier approx.	24.9	0.1 (SE fit)
	EOM	25.2	N/A
	MFF	23.9	0.12 (SE fit)
simulations			
SERF	CAMPARI (all-atom)	25.9	0.8 (SD of replicate means)
	mPiPi (coarse-grained)	25.4	0.1 (SD of conformer means)
SERF (+ $r(U)_{29}$)*	mPiPi	22.9	
SERF (+ TAR)*	mPiPi	22.1	0.2 (SD of conformer means)

* R_G calculations for SERF in complex were performed on the protein chain only; the RNA chain was ignored.

Acronyms: AFRC – analytical Flory random coil[32]; ALBATROSS – deep learning-based predictor[33]; EOM – ensemble optimization method[34]; MFF – molecular form factor[35]; SE – standard error; SD – standard deviation.

Table S3: Dissociation constants from experiments and simulations.

Protein	RNA	K_D (μM)†	error‡‡
experiments			
SERF	TAR	0.67	0.04
SERF	$r(U)_{30}$	1.9	0.2
coarse-grained simulations			
SERF	TAR	1.2	0.1
SERF	$r(U)_{29}$	4.1	0.6
$SERF_{1\text{-}34}$	TAR	1.1	0.1
$SERF_{1\text{-}34}$	$r(U)_{29}$	3.6	0.7

†Dissociation constants from coarse-grained simulations are not absolute values; a correction factor of 10 was introduced into the analysis workflow for easier comparison between experiments and simulations.

‡From experiments, the reported error is the standard deviation of the fit from the covariance matrix; the error from simulations is the standard error of the mean calculated from K_D values across independent replicates.

Table S4: Masses and collision cross-sections (CCSs) of all SERF, TAR, and SERF-TAR complex ions detected by IM-MS with nitrogen or helium carrier gas.

ID	Ion Mass	Ion Charge (z)	CCS, N_2 (nm^2)	CCS, N_2 S.D.	CCS, He (nm^2)	CCS, He S.D.
**SERF**	7949.4 (+/- 0.2 Da)	13	24.52	0.02	21.11	0.02
		12	23.16	0.05	19.92	0.04
		11	21.80	0.01	18.73	0.01
		10	20.67	0.00	17.74	0.00
		9	19.47	0.01	16.69	0.01
		8	18.27	0.01	15.64	0.01
		7	13.15	0.02	11.15	0.02
		7	14.27	0.04	12.13	0.03
		7	15.49	0.01	13.20	0.01
		7	17.32	0.01	14.80	0.01
		6	11.67	0.03	9.85	0.02
		6	12.13	0.08	10.26	0.07
		6	14.21	0.10	12.08	0.09
		5	11.23	0.01	9.47	0.01
**2SERF**	15898 (+/- 1 Da)	8	17.34	0.03	14.82	0.03
		8	18.66	0.09	15.97	0.08
		6	12.59	0.10	10.66	0.08
**TAR**	9290.7 (+/- 0.4 Da)	5	10.53	0.01	8.85	0.01
		5	11.02	0.06	9.29	0.05
		4	9.95	0.02	8.35	0.02
		4	10.29	0.06	8.64	0.05
**SERFTAR**	17240 (+/- 2 Da)	11	26.03	0.19	22.43	0.16
		11	26.75	0.18	23.06	0.16
		10	18.70	0.07	16.01	0.06
		9	17.80	0.02	15.22	0.02
		8	17.14	0.06	14.64	0.05
		7	16.62	0.05	14.19	0.04
		6	16.13	0.04	13.76	0.03
**2SERFTAR**	25189 (+/- 1 Da)	11	23.20	0.12	19.96	0.10
		11	24.22	0.10	20.85	0.09
		10	22.38	0.11	19.23	0.09
		9	21.83	0.09	18.76	0.08

Table S5: Alignment tensor derived from the analysis of residual dipolar couplings (RDCs) measured on TAR alone and in a non-saturated TAR-SERF 1:1 complex.

System	Helix	A_a (10^{-4})†	A_r (10^{-4})†	α (°)	β (°)	γ (°)	Scalar Product
TAR	1	4.79	0.27	35	38	-76	0.24
	2	8.19	0.53	-50	17	44	
TAR-SERF	1	3.25	1.55	-134	170	-67	0.82
	2	8.34	0.59	-27	168	19	

†A_a – axial component of the alignment tensor; A_r – rhombicity of the alignment tensor

REFERENCES

1. Marley, J., Lu, M. & Bracken, C. A method for efficient isotopic labeling of recombinant proteins. *J Biomol NMR* 20, 71–75 (2001).
2. Sahu, D., Bastidas, M. & Showalter, S. A. Generating NMR chemical shift assignments of intrinsically disordered proteins using carbon-detected NMR methods. *Anal Biochem* 449, 17–25 (2014).
3. Tamiola, K., Acar, B. & Mulder, F. A. A. Sequence-specific random coil chemical shifts of intrinsically disordered proteins. *J Am Chem Soc* 132, 18000–18003 (2010).
4. Lawrence, C. W. & Showalter, S. A. Carbon-detected 15N NMR spin relaxation of an intrinsically disordered protein: FCP1 dynamics unbound and in complex with RAP74. *Journal of Physical Chemistry Letters* 3, 1409–1413 (2012).
5. Delaglio, F. *et al.* NMRPipe: A multidimensional spectral processing system based on UNIX pipes. *J Biomol NMR* 6, 277–293 (1995).
6. Skinner, S. P. *et al.* CcpNmr AnalysisAssign: a flexible platform for integrated NMR analysis. *J Biomol NMR* 66, 111–124 (2016).
7. Dosset, P., Hus, J.-C., Blackledge, M. & Marion, D. Efficient analysis of macromolecular rotational diffusion from heteronuclear relaxation data. *J Biomol NMR* 16, 23–28 (2000).
8. Salmon, L., Bascom, G., Andricioaei, I. & Al-Hashimi, H. M. A general method for constructing atomic-resolution RNA ensembles using NMR residual dipolar couplings: The basis for interhelical motions revealed. *J Am Chem Soc* 135, 5457–5466 (2013).
9. Jensen, M. R., Ortega-Roldan, J. L., Salmon, L., Van Nuland, N. & Blackledge, M. Characterizing weak protein-protein complexes by NMR residual dipolar couplings. *European Biophysics Journal* 40, 1371–1381 (2011).
10. Lalmansingh, J. M., Keeley, A. T., Ruff, K. M., Pappu, R. V. & Holehouse, A. S. SOURSOP: A Python Package for the Analysis of Simulations of Intrinsically Disordered Proteins. *J Chem Theory Comput* 19, 5609–5620 (2023).
11. McGibbon, R. T. *et al.* MDTraj: A Modern Open Library for the Analysis of Molecular Dynamics Trajectories. *Biophys J* 109, 1528–1532 (2015).
12. Battiste, J. L. & Wagner, G. Utilization of Site-Directed Spin Labeling and High Resolution Heteronuclear Nuclear Magnetic Resonance for Global Fold Determination of Large Proteins with Limited Nuclear Overhauser Effect Data. *Biochemistry* 39, 5355–5365 (2000).
13. Meng, W., Lyle, N., Luan, B., Raleigh, D. P. & Pappu, R. V. Experiments and simulations show how long-range contacts can form in expanded unfolded proteins with negligible secondary structure. *Proceedings of the National Academy of Sciences* 110, 2123–2128 (2013).

14. Schneidman-Duhovny, D., Hammel, M., Tainer, J. A. & Sali, A. Accurate SAXS Profile Computation and its Assessment by Contrast Variation Experiments. *Biophys J* 105, 962–974 (2013).
15. Hopkins, J. B., Gillilan, R. E. & Skou, S. *BioXTAS RAW* : improvements to a free opensource program for small-angle X-ray scattering data reduction and analysis. *J Appl Crystallogr* 50, 1545–1553 (2017).
16. Humphrey, W., Dalke, A. & Schulten, K. VMD: Visual molecular dynamics. *J Mol Graph* 14, 33–38 (1996).
17. Pettersen, E. F. *et al.* <scp>UCSF ChimeraX</scp> : Structure visualization for researchers, educators, and developers. *Protein Science* 30, 70–82 (2021).
18. Fossat, M. J. & Pappu, R. V. *q* -Canonical Monte Carlo Sampling for Modeling the Linkage between Charge Regulation and Conformational Equilibria of Peptides. *J Phys Chem B* 123, 6952–6967 (2019).
19. Fossat, M. J., Posey, A. E. & Pappu, R. V. Quantifying charge state heterogeneity for proteins with multiple ionizable residues. *Biophys J* 120, 5438–5453 (2021).
20. Fossat, M. J., Posey, A. E. & Pappu, R. V. Uncovering the Contributions of Charge Regulation to the Stability of Single Alpha Helices**. *ChemPhysChem* 24, (2023).
21. Dunlap, T. B. *et al.* Stoichiometry of the Calcineurin Regulatory Domain–Calmodulin Complex. *Biochemistry* 53, 5779–5790 (2014).
22. Jarmoskaite, I., Alsadhan, I., Vaidyanathan, P. P. & Herschlag, D. How to measure and evaluate binding affinities. *Elife* 9, (2020).
23. Stow, S. M. *et al.* An Interlaboratory Evaluation of Drift Tube Ion Mobility–Mass Spectrometry Collision Cross Section Measurements. *Anal Chem* 89, 9048–9055 (2017).
24. Kurulugama, Ruwan. T., Darland, E., Kuhlmann, F., Stafford, G. & Fjeldsted, J. Evaluation of drift gas selection in complex sample analyses using a high performance drift tube ion mobility-QTOF mass spectrometer. *Analyst* 140, 6834–6844 (2015).
25. Polasky, D. A., Dixit, S. M., Fantin, S. M. & Ruotolo, B. T. CIUSuite 2: Next-Generation Software for the Analysis of Gas-Phase Protein Unfolding Data. *Anal Chem* 91, 3147–3155 (2019).
26. Joseph, J. A. *et al.* Physics-driven coarse-grained model for biomolecular phase separation with near-quantitative accuracy. *Nat Comput Sci* 1, 732–743 (2021).
27. Alston, J. J., Soranno, A. & Holehouse, A. S. Conserved molecular recognition by an intrinsically disordered region in the absence of sequence conservation. *bioRxiv* (2023) doi:10.1101/2023.08.06.552128.
28. Cubuk, J. *et al.* The disordered N-terminal tail of SARS-CoV-2 Nucleocapsid protein forms a dynamic complex with RNA. *Nucleic Acids Res* 52, 2609–2624 (2024).
29. Tesei, G., Schulze, T. K., Crehuet, R. & Lindorff-Larsen, K. Accurate model of liquid–liquid phase behavior of intrinsically disordered proteins from optimization of single chain properties. *Proceedings of the National Academy of Sciences* 118, (2021).
30. Jost Lopez, A., Quoika, P. K., Linke, M., Hummer, G. & Köfinger, J. Quantifying Protein Protein Interactions in Molecular Simulations. *Journal of Physical Chemistry B* 124, 4673–4685 (2020).
31. Ganguly, P. & Van Der Vegt, N. F. A. Convergence of sampling Kirkwood-Buff integrals of aqueous solutions with molecular dynamics simulations. *J Chem Theory Comput* 9, 1347–1355 (2013).

32. Alston, J. J., Ginell, G. M., Soranno, A. & Holehouse, A. S. The Analytical Flory Random Coil Is a Simple-to-Use Reference Model for Unfolded and Disordered Proteins. *J Phys Chem B* 127, 4746–4760 (2023).
33. Lotthammer, J. M., Ginell, G. M., Griffith, D., Emenecker, R. J. & Holehouse, A. S. Direct prediction of intrinsically disordered protein conformational properties from sequence. *Nat Methods* 21, 465–476 (2024).
34. Bernadó, P., Mylonas, E., Petoukhov, M. V., Blackledge, M. & Svergun, D. I. Structural characterization of flexible proteins using small-angle X-ray scattering. *J Am Chem Soc* 129, 5656–5664 (2007).
35. Riback, J. A. *et al.* Innovative scattering analysis shows that hydrophobic disordered proteins are expanded in water. *Science (1979)* 358, 238–241 (2017).
36. Schneidman-Duhovny, D., Hammel, M. & Sali, A. FoXS: a web server for rapid computation and fitting of SAXS profiles. *Nucleic Acids Res* 38, W540–W544 (2010).
